# Estrogen receptor signaling drives immune evasion and immunotherapy resistance in HR^+^ breast cancer

**DOI:** 10.1172/JCI193153

**Published:** 2025-11-25

**Authors:** José Ángel Palomeque, Gabriel Serra-Mir, Sandra Blasco-Benito, Helena Brunel, Pau Torren-Duran, Iván Pérez-Núñez, Chiara Cannatá, Laura Comerma, Silvia Menendez, Sonia Servitja, Tamara Martos, Maria Castro, Rodrigo L. Borges, Joanna I. López-Velazco, Sara Manzano, Santiago Duro-Sánchez, Joaquín Arribas, María M. Caffarel, Ander Urruticoechea, José A. Seoane, Lluis Morey, Joan Albanell, Toni Celià-Terrassa

**Affiliations:** 1 Cancer Research Program, Hospital del Mar Research Institute (HMRI), Barcelona, Spain.; 2 Pathology Department, Hospital del Mar, Barcelona, Spain.; 3 Medical Oncology Department, Hospital del Mar, Barcelona, Spain.; 4 Universitat Pompeu Fabra, Barcelona, Spain.; 5 Centro de Investigación Biomédica en Red de Oncología (CIBERONC-ISCIII), Madrid, Spain.; 6 Sylvester Comprehensive Cancer Center, University of Miami Miller School of Medicine, Miami, Florida, USA.; 7 Biogipuzkoa (formerly Biodonostia) Health Research Institute, San Sebastian, Spain.; 8 Preclinical and Translational Research Program, Vall d’Hebron Institute of Oncology (VHIO), Barcelona, Spain.; 9 Institució Catalana de Recerca i Estudis Avançats (ICREA), Barcelona, Spain.; 10 IKERBASQUE, Basque Foundation for Science, Bilbao, Spain.; 11 Gipuzkoa Cancer Unit, OSI Donostialdea – Onkologikoa Foundation, San Sebastian, Spain.; 12 Cancer Computational Biology Group. Vall d’Hebron Institue of Oncology, Barcelona, Spain.; 13 Department of Human Genetics, University of Miami Miller School of Medicine, Miami, Florida, USA.

**Keywords:** Immunology, Oncology, Breast cancer, Cancer immunotherapy, Molecular biology

## Abstract

Hormone receptor–positive (HR^+^) breast cancers (BCs) are typically “immune-cold,” poorly immune-infiltrated tumors that do not respond to immune-checkpoint blockade (ICB) therapies. Using clinical data, we report that estrogen receptor α (ERα) signaling was associated with immunosuppressive pathways and a lack of response to ICB in patients with HR^+^ BC. In this study, we validated ER-mediated immunosuppression by engineering and modulating the ER in preclinical models in vitro, in vivo, and ex vivo. Mechanistically, we found that ERα hijacked LCOR, a nuclear receptor corepressor, thereby preventing LCOR’s function in the induction of tumor immunogenicity and immune infiltration, which is normally observed in the absence of ERα, such as in ER^–^ BC. In HR^+^ BC, we demonstrate that the molecular disruption of LCOR and ERα interaction using anti-ER therapies or using a mutant of the LCOR nuclear receptor–binding domain (LSKLL into LSKAA) that does not interact with ERα, restored the immunogenic functions of LCOR. Remarkably, the LCOR-ERα disruption converted HR^+^ BC immune-cold tumors into immune-hot tumors responsive to ICB by increased antigen presentation machinery expression, immune infiltration, T cell recognition, and T cell–mediated killing. In conclusion, ERα inhibition and the disruption of LCOR-ERα interaction represent a therapeutic strategy and an opportunity to elicit immunotherapeutic benefit in patients with HR^+^ BC.

## Abstract 2

This study shows for the first time why HR+BC (most common BC subtype) is resistant to immunotherapy. We reveal a therapeutic strategy that overcomes resistance.

## Graphical abstract

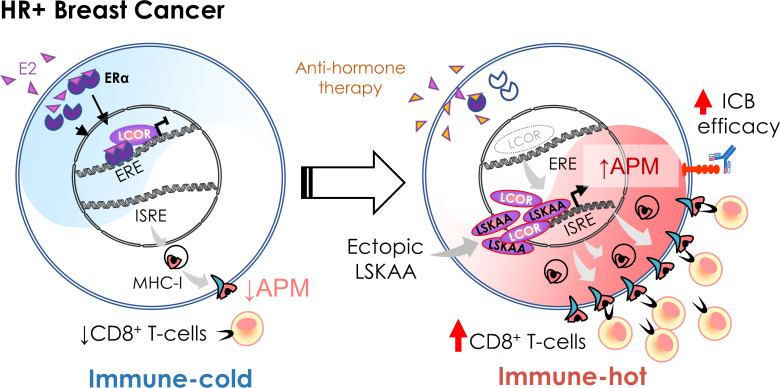

## Introduction

Hormone receptor–positive (HR^+^) breast cancer (BC) constitutes 70%–75% of breast tumors, characterized by the expression of the estrogen receptor α (ER^+^) and the progesterone receptor (PR^+^) (1, 2). This molecular subtype presents high 5-year survival rates for local disease with adjuvant endocrine therapies as standard of care (SOC) targeting ERα signaling. Still, the outcome for patients with HR^+^ BC drastically decrease for those with recurrent disease and those with advanced metastatic disease, which occur in 20%–30% of patients (3) and ultimately account for most BC deaths (4, 5) pinpointing the clinical need for innovative therapies.

Immune-checkpoint blockade (ICB) immunotherapy has emerged as an effective treatment for different cancer types, boosting antitumor immune responses. In BC, ICB has only been approved for triple-negative BC (TNBC) (6, 7) despite attempts to apply this therapy in all subtypes in different clinical trials. HR^+^ BC tumors are the least responsive and are typically “immune-cold” tumors with low immune infiltration compared with the other subtypes (8–11). Weak responses to ICB have been observed in the neoadjuvant setting when combined with chemotherapy (8, 12), and responses are virtually absent in patients with advanced HR^+^ BC (13–17). Several studies have linked the HR^+^ BC immune-cold phenotype to different clinicopathological features of HR^+^ tumors: low mutational burden (18), low programmed cell death ligand 1 (PD-L1) expression (19), low number of tumor-infiltrating lymphocytes (TILs) (20, 21), and enrichment of immunosuppressive populations (22, 23). Remarkably, the clinical trials in HR^+^ BC — KEYNOTE-756 and CheckMate 7FL — have correlated high levels of ERα and PR percentage with a lack of response to ICB therapies (24–26). Other clinical studies have shown a negative correlation between ERα expression, immune infiltration, and pathways that play a central role in the ICB response: IFN signaling, antigen presentation machinery (APM), and tumor inflammation (10, 27, 28). Intriguingly, these observations suggest a connection between ERα signaling and the immune response as a potential molecular mechanism that remains poorly explored.

ERα activity is controlled by several cofactors that comprise transcription factors, pioneer factors, epigenetic modifiers, chromatin remodelers, coactivators, and corepressors (29, 30). Nuclear receptor corepressors comprise a family of proteins that inhibit nuclear receptor–associated transcription through different enzymatic activities. Nuclear receptor corepressors are subdivided into LXXH/IIXXXI/L-containing motifs and LXXLL-containing motifs, the second corepressors recruited to chromatin only upon nuclear receptor binding with ligand (31). ERα’s LXXLL corepressors are RIP140 and the Ligand receptor CoRepressor (LCOR) (30, 31). LCOR interacts with nuclear receptors through the LSKLL domain and participates in the ER signaling repression through the recruitment of histone deacetylation (HDAC) enzymes and C-terminal binding proteins (CtBP) (32, 33). However, LCOR is a conserved protein and contains other domains including a DNA-binding domain helix-turn-helix (HTH) (34–36). The HTH domain is the most conserved part of the protein, and we reported a transcriptional activator function in TNBC that results in the induction of antigen presentation (37, 38). Therefore, in TNBC, which is ER^–^, LCOR binds gene loci involved in APM and IFN-stimulatory response elements (ISREs) that activate gene transcription. This nuclear receptor–independent function of LCOR increases tumor immunogenicity and visibility to the immune system, which has been shown to be beneficial for immunotherapy in preclinical models, leading to complete eradication of TNBC tumors (37).

Here, we demonstrate that in HR^+^ BC, ERα signaling drove immunosuppression and immunotherapy resistance, in part by hijacking LCOR’s immunogenic functions. Therefore, genetic and molecular disruption of LCOR and ERα interaction restores LCOR genomic localization in APM regions and thus its ability to regulate APM gene expression. Targeting and preventing the LCOR-ERα complex through anti-ER therapies or genetic mutation of the LCOR nuclear receptor sequence (LSKAA) converts HR^+^ BC cold tumors into hot tumors. This molecular strategy represents an innovative therapeutic opportunity to convert HR^+^ BC tumors into hot, immunogenically eligible tumors that will respond to immune-based therapies, presenting a therapeutic option for advanced HR^+^ BC that is currently lacking.

## Results

### ER signaling is associated with immunotherapy resistance and reduced immunogenic pathways in patients.

**Figure 1:**
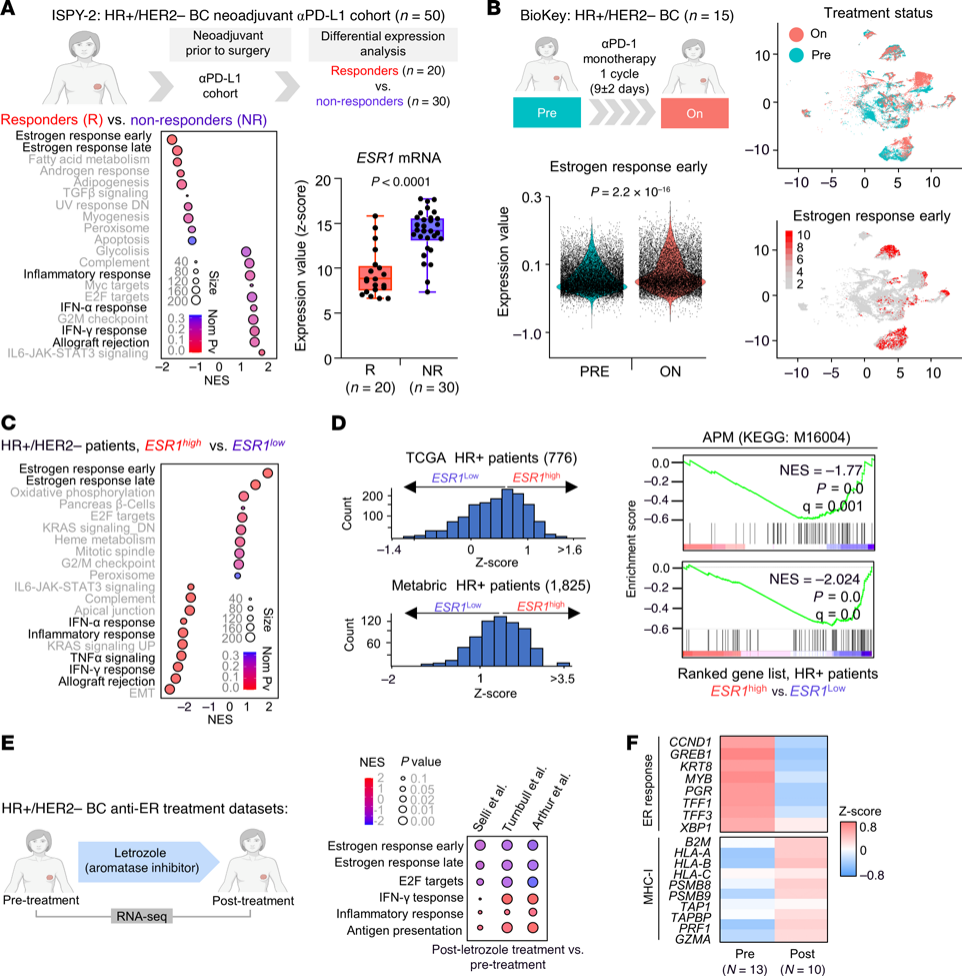
ER signaling is associated with immunotherapy resistance and reduced immunogenic pathways in patients. (**A**) GSEA of ranked transcripts for HR^+^HER2^–^ BC patients classified as Rs (pCRs) versus NRs to the neoadjuvant anti–PD-L1/olaparib/Nab-paclitaxel arm of the ISPY-2 clinical trial (12). Data were ranked according to the normalized enrichment score (NES). Bubble size represents signature gene size, and the color scale depicts the nominal *P* value. Analysis of *ESR1* mRNA levels in R and NR patients. Box plot represents the IQR with individual points. The adjusted *P* value was determined by Benjamini-Hochberg correction. αPD-L1, anti–PD-L1. (**B**) Analysis of scRNA-Seq of tumor cells from HR^+^ patients treated with anti–PD-1 (*n* = 15) from the BioKey clinical trial (45). Violin plot shows the estrogen response early signature (hallmarks, M5906), calculated using the Seurat function for patients before (Pre) and after (On) 1 cycle of anti–PD-1 monotherapy. Uniform manifold approximation and projection (UMAP) representation of time points (On and Pre) and overlapping signature expression with the color scale expression score. The *P* value shown was determined by Wilcoxon’s paired test for Pre versus On. (**C**) GSEA of ranked transcripts comparing *ESR1*^hi^ versus *ESR1*^lo^ HR^+^HER2^–^ BC patients from the METABRIC BC public dataset. Data were ranked according to the NES. Bubble size represents the signature gene size, and the color scale depicts the nominal *P* value. (**D**) GSEA of APM (KEGG code: M16004) comparing *ESR1*^hi^ versus *ESR1*^lo^ patients ranked from *ESR1* mRNA median expression of HR^+^HER2^–^ group in METABRIC and TCGA datasets. Distribution of *ESR1* mRNA levels across patients is shown as a *z* score. (**E**) GSEA of hallmark transcripts (estrogen response early, estrogen response late, E2F targets, IFN-γ response, and inflammatory response) and KEGG legacy (APM) comparing transcriptomes of patients before treatment versus 2 weeks after letrozole therapy from specified datasets (46–48). Bubble size represents the nominal *P* value, and color depicts the NES. (**F**) Heatmap of the expression (*z* score) of genes related to an estrogen response and MHC-I pathways for patients before treatment and 2 weeks after letrozole therapy.

To explore the molecular mechanisms of immunotherapy resistance in HR^+^ tumors, we analyzed the transcriptomic profiles of published immunotherapy clinical trials for HR^+^ BC. Gene set enrichment analysis (GSEA) of responders (Rs) to therapy (referred to as a pathologic complete response [pCR]) and nonresponders (NRs) to anti–PDL1 combinations from the second arm of the ISPY-2 Clinical trial (12), scored the early and late estrogen responses as the top enriched pathways in NR patients (Figure 1A). In contrast, R patients showed enrichment of the inflammatory, IFN-γ, and IFN-α response pathways, which are related to immunogenic responses (39, 40). As estrogen response signatures report ERα activity (*ESR1*) (41), we further assessed the levels of *ESR1* mRNA and observed significantly higher expression of *ESR1* in NR patients (Figure 1A). To further analyze ERα signaling dynamics during therapy-mediated immune pressure, we evaluated the estrogen response using single-cell RNA-Seq available data from patients with HR^+^ BC before (pretreatment) and during (on-treatment) therapy consisting of 1 cycle of anti–PD-1 treatment (42). Our analysis of the ER pathway showed how on-treatment patients had higher levels of the estrogen response signature than did pretreatment patients (Figure 1B), suggesting a positive selection of ER^+^ cells able to escape anti–PD-1 treatment.

Using large clinical BC transcriptomics datasets (The Cancer Genome Atlas [TCGA] and METABRIC), only patients with *ESR1*^lo^ expression had higher levels of immune-related pathways like IFN, the inflammatory response, and APM signatures than did those with *ESR1*^hi^ expression (Figure 1, C and D), suggesting a stronger immunogenic profile in *ESR1*^lo^ patients. We observed the same result when we compared patients with ER^+^ BC with those with ER^–^ BC and BC cell lines from the Cancer Cell Line Encyclopedia (CCLE), which showed enrichment of the IFN and APM signatures in ER^–^ BC compared with ER^+^ BC (Supplemental Figure 1, A and B; supplemental material available online with this article; https://doi.org/10.1172/JCI193153DS1). In addition, the immune cell deconvolution analysis of the METABRIC ER^+^ BC dataset showed that the low *ESR1* mRNA cluster (C1) was highly infiltrated and high *ESR1* mRNA levels in the poorly infiltrated cluster (C3) (Supplemental Figure 1C). Similarly, using the BioKey scRNA-Seq dataset, almost no overlap between MHC-I and *ESR1* expression was observed (Supplemental Figure 1D), and the comparison between ER^–^ and ER^+^ cells in the same patient showed higher expression of the MHC-I genes in the ER^–^ tumor compartment (Supplemental Figure 1E).

Moreover, to explore whether the clinical intervention on ERα activity can affect tumor immunogenic pathways, we interrogated 3 datasets before and after anti-ER aromatase inhibitor letrozole (43–45). We observed increased expression of immunogenic signatures (APM, IFN response, and inflammatory response signatures) after letrozole alongside the reduction of ER activity (Figure 1E and Supplemental Figure 1, F and G). Remarkably, MHC-I expression showed an inverse correlation with ER targets after treatment (Figure 1F).

Overall, the corollary of clinical dataset analyses indicated an inverse correlation of ER signaling with immune responses, thus compelling us to focus our mechanistic rationale of immunotherapeutic resistance of HR^+^ BC on ERα signaling.

### Preclinical modeling of ER-driven, immune-evasive BC tumors.

**Figure 2:**
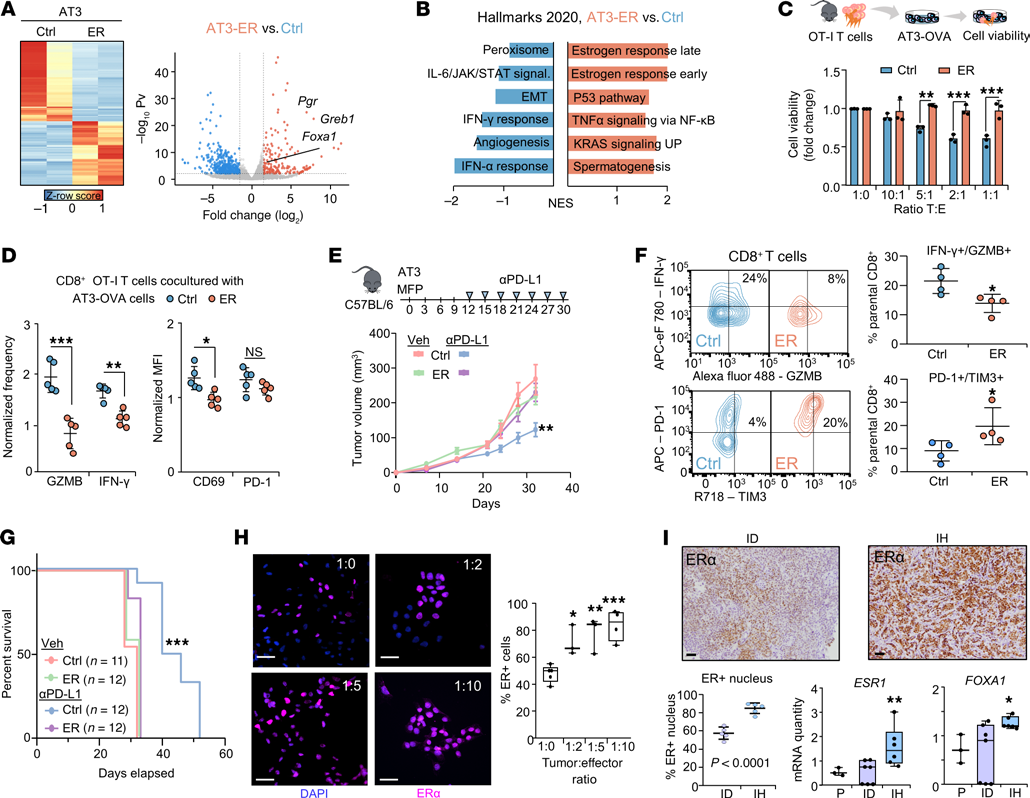
Preclinical modeling of ER-driven, immune-evasive BC cold tumors. (**A**) Heatmap of unsupervised hierarchical clustering analysis from RNA-Seq of AT3 vector control (Ctrl) and AT3-ER overexpression (ER). *n* = 2 independent biological replicates. The *Z* row score represents read counts. Volcano plot shows transcriptomics analysis from AT3-ER versus AT3 control RNA-Seq. Estrogen target genes are highlighted. (**B**) GSEA analysis of upregulated (red) and downregulated (blue) pathways (MSigDB hallmarks 2020) comparing AT3-ER versus AT3 control transcriptomes. Pathways are ranked by the NES. (**C**) CTL assay of AT3-OVA control and ER-OE cells treated with INF-**γ** (1 μg/mL) at different ratios of tumor (T) and effector (E) T cells isolated from OT-I mice. (**D**) Flow cytometric analysis of cytotoxic (GZMB^+^ and IFN-γ^+^) and activation (CD69^+^ and PD-1^+^) markers from CD8^+^OT-1^+^ T cells after coculturing with AT3-OVA control or ER-OE cells. *n* = 5 independent biological replicates. (**E**) Growth curves of AT3 and AT3-ER tumors in C57BL/6 mice. Once tumors reached 0.5 × 0.5 cm^2^ in size, mice were treated twice a week with vehicle or 7.5 mg/kg anti–PD-L1. *n* = 12 tumors per condition. (**F**) Flow cytometric analysis of cytotoxic (IFN-γ^+^GZMB^+^) or exhausted (PD-1^+^TIM3^+^) infiltrating CD8^+^ T cells from AT3 control or ER-OE tumors. *n* = 4 independent biological replicates. (**G**) Survival curves for AT3 control tumor– and ER tumor–bearing mice treated with vehicle or 7.5 mg/kg anti–PD-L1. (**H**) Representative images of ERα staining of mixed AT3-OVA control plus AT3-OVA-ER–OE cells. Scale bars: 50 μm. Box plot shows quantification of the ER^+^ percentage across different conditions of tumor/effector ratios and represents the IQR with individual points. (**I**) IHC images of ERα^+^ MCF-7 tumors from NSG mice transfused with human PBMCs (IH) or PBS (ID). Scale bars: 100 μM. Quantification graph showing the percentage of ERα positivity in the images. *n* = 5 biological replicates. RT-qPCR analysis of MCF-7 parental cells and MCF-7 tumors in ID and IH settings. *n* = 6 mice. Box plots represent the IQR with individual points. The *P* value from statistical analysis of ID versus IH is shown. Data represent the mean ± SEM (**C**–**F**). *P < 0.05, **P < 0.01, and ***P < 0.001, by 2-way ANOVA (**C**, **D**, and **F**), 1-way ANOVA (**H** and **I**), mixed-design ANOVA (**E**), and log-rank (Mantel-Cox) test (**G**). In **D** and **F**, cells were gated as shown in Supplemental Figure 8C. Veh, vehicle.

To validate the influence of ERα activity on tumor immunology in mice, we engineered a syngeneic BC mouse model of ERα ectopic expression using AT3 cells, which are typically ER^–^ cells but are derived from luminal-like PyMT tumors. The engineered AT3-ER cells showed correct ERα expression and nuclear localization by Western blotting and immunofluorescence (IF) (Supplemental Figure 2A). Transcriptomics profiles of AT3-ER overexpression showed canonical ERα functionality with an increase in the classical ER targets and cofactors *Foxa1*, *Greb1*, and *Pgr*; thus validating the ER biology of our model (46, 47) (Figure 2A and Supplemental Figure 2B). Analysis of differential expression between AT3-ER and control AT3 revealed enrichment of the estrogen response and downregulation of the IFN response and APM (Figure 2B and Supplemental Figure 2C), consistent with our observations from the clinical data previously mentioned (Figure 1).

After assessing the validity of the AT3-ER model by transcriptomics profiles, we next performed functional assays to test the immune evasion mediated by ERα. We performed in vitro cytotoxic T lymphocyte (CTL) assays of AT3-OVA control and AT3-OVA-ER–overexpressing (OE) cells with OT-I T cells, whose TCR specifically recognizes the OVA peptide SIINFEKL presented by H2K^b^ (48). AT3-OVA-ER cells showed less T cell–mediated killing than did AT3-OVA control cells in the different tumor/effector (T/E) ratios (Figure 2C), along with a reduction of T cell activation (CD69) and cytotoxic markers (IFN-γ and granzyme B [GZMB]) (Figure 2D). Next, AT3-ER-OE cells implanted into C57BL/6 immunocompetent (IC) mice exhibited no response to anti–PD-L1 therapy compared with AT3 control tumors in vivo (Figure 2E). Flow cytometric analysis after treatment showed a lower proportion of activated T cells (IFN-γ^+^GZMB^+^) and a higher proportion of exhaustion markers (TIM3^+^PD-1^+^) in AT3-ER tumors (Figure 2F), consistent with their lack of response and reduced survival compared with control (ER^–^) tumors (Figure 2G). Next, we performed an assay to determine whether modulating E2 levels could impair ER-mediated immune evasion (49). In vitro, we found that E2 depletion resulted in enhanced T cell killing of AT3-OVA-ER cells (Supplemental Figure 2D). In vivo, E2^lo^ (withdrawing E2 supplementation) favored a response to anti–PD-L1 therapy (Supplemental Figure 2E) in an IC setting through a significant reduction of the estrogen response (Supplemental Figure 2F).

To model the ER^+^ tumor positive selection observed in patients upon immune pressure (Figure 1B), we conducted CTL assays, mixing AT3-OVA control and AT3-OVA-ER–OE cells. After coculturing with OT-I T cells, only ER^+^ cells survived the immune attack, highlighting their ability to surpass the immune pressure (Figure 2H). In order to prove this in human preclinical settings, we used human MCF-7 BC cells in immune-humanized (IH), immunodeficient (ID) NSG mice. After mammary fat pad (MFP) MCF-7 transplantation and establishment of tumors, mice were randomized to the ID group (tail-vein injection of vehicle) or the IH group (tail vein administration of 10 × 10^6^ PBMCs from healthy donors). As expected, we found that IH mice had reduced tumor growth compared with ID mice (Supplemental Figure 2G) due to the immune pressure of PBMCs. After 3 weeks, IH tumors showed increased ERα expression compared with ID tumors (Figure 2I), as measured by IHC and estrogen response–related transcript expression (*ESR1* and *FOXA1*) (Figure 2I), consistent with the selective pressure effect. Thus, MCF-7 cells with higher ERα expression had a greater ability to surpass the immune attack in this humanized model. This same model also showed the expected response to ERα inhibition when mice that were treated in vitro with tamoxifen showed increased proinflammatory pathways, such as TNF-α signaling and IFN response pathways (Supplemental Figure 2H). Overall, our preclinical functional tests demonstrate the immune-evasive effects of ERα signaling in HR^+^ BC.

### LCOR immunogenic effects are abrogated by ERα in HR^+^ BC.

**Figure 3:**
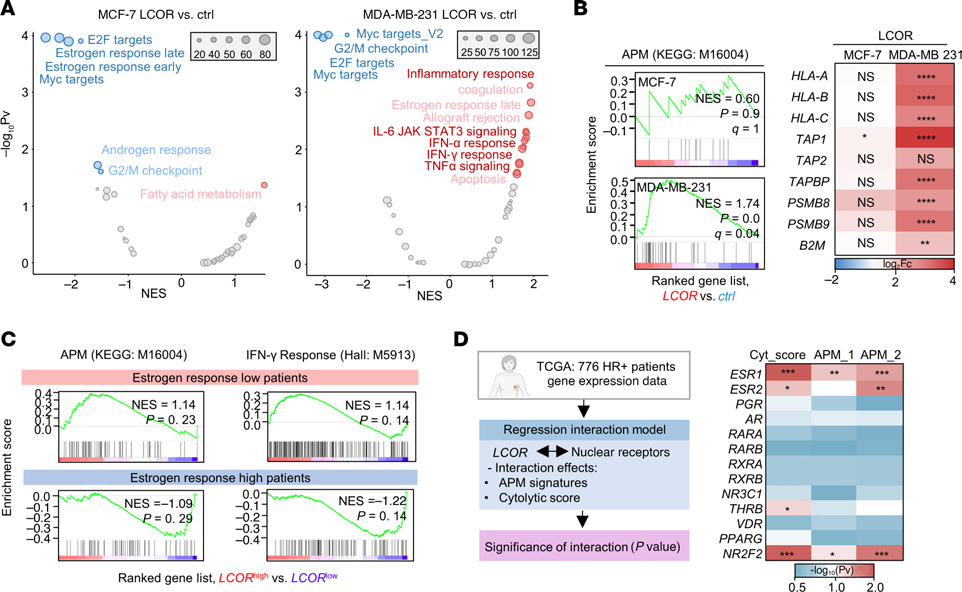
LCOR immunogenic effects are abrogated by ERα in HR^+^ BC. (**A**) Volcano plot showing ranked gene signatures from GSEA analysis of LCOR-OE versus control cells following RNA-Seq of MDA-MB-231 and MCF-7 cells. Data are ranked on the basis of –log_10_ *P* value (Pv) and NES values. The size of the dataset is represented. (**B**) GSEA of APM (KEGG: M16004) comparing LCOR-OE versus control MDA-MB-231 and MCF-7 cells. Heatmap of RNA-Seq analysis of MHC-I genes in LCOR-OE and control MCF-7 and MDA-MB-231 cells. (**C**) GSEA of the Scan-B BC clinical dataset (53). HR^+^ patients (*n* = 2,423) were stratified by *ESR1/PGR/NR2F2* expression terciles in high (*n* = 824) and low (*n* = 800). Within each group, patients were ranked according to *LCOR* median expression. Groups were interrogated for the APM signature (KEGG: M16004) and IFN-γ response (hallmarks [Hall], M5913). (**D**) Computational interaction regression model in TCGA HR^+^ BC clinical data (*n* = 776 patients). The interaction model was calculated according to Score~geneA*geneB, where gene A is *LCOR*, gene B represents the different nuclear receptors, and the score represents pathways assessed as the APM (KEGG: M16004), MHC-I, and cytolytic scores. Heatmap represents the log_10_ *P* value of the statistic applied to calculate the interaction.

We have previously shown that the ligand-dependent nuclear receptor corepressor, LCOR, is a potent inducer of immunogenicity in TNBC (37, 38). Here, we aimed to study whether LCOR immunomodulatory functions are intersected by ERα in ER^+^ tumors cells. To have a comparative analysis of the LCOR effects in HR^+^ models, we performed RNA-Seq of LCOR-OE in HR^+^ BC (MCF-7) and TNBC (MDA-MB-231) cells (Supplemental Figure 3, A and B). Intriguingly, principal component analysis (PCA) revealed that LCOR-OE and control samples clustered closely in MCF-7 cells, whereas they diverged in MDA-MB-231 cells (Supplemental Figure 3C), suggesting that LCOR function may have been restricted in the HR^+^ BC context. The unsupervised clustering analysis of samples showed 7,418 differentially expressed genes (DEGs) in MDA-MB-231 LCOR-OE samples and 2,562 DEGs in MCF-7 LCOR–OE samples (Supplemental Figure 3, A and D) with only 1,435 (16%) DEGs in common, although this included divergent upregulated and downregulated genes when comparing both cell types. Only in MDA-MB-231 cells did LCOR overexpression have the expected induction of inflammatory responses, IFN-α/IFN-γ signaling, and APM signatures (Figure 3, A and B). However, in MCF-7 cells LCOR failed to upregulate any of the expected immunogenic pathways, despite the observed downregulation of an estrogen response (Figure 3, A and B). Moreover, ChIP enrichment analysis (ChEA) ranked *ESR1* as predominant factor controlling the expression of LCOR-downregulated transcripts from the MCF-7 RNA-Seq data (Supplemental Figure 3E), suggesting that, in the HR^+^ BC context, LCOR functioned as a nuclear receptor corepressor governed by the activity of ERα.

To validate whether these findings are also reflected in clinical disease, we used the integrative public BC dataset from the Scan-B clinical trial (50). We performed GSEA in subgroups of patients classified as having a low (*n* = 800 patients) or high (*n* = 824 patients) estrogen response, ranked according to *LCOR* median expression. The analysis validated that *LCOR* stratification was associated with increased IFN and APM signatures in the *ESR1*^lo^ patients, but not in the *ESR1*^hi^ patients, reflecting the effect of ERα on LCOR function, as seen in the MCF-7 LCOR–OE cells’ transcriptome (Figure 3C). In order to extend this correlation without the effect of stromal cells, we also analyzed different cells lines in the CCLE, which shows how LCOR expression does not correlate with APM in ER^+^ BC cell lines but does correlate with ER^–^ BC cells (Supplemental Figure 3F).

HR^+^ tumors had higher levels of ERα (*ESR1*, but not *ESR2*), the PR, as well as other nuclear receptors compared with TNBC tumors, that could interact with LCOR (Supplemental Figure 3G). In order to dissect which nuclear receptors affect the immunogenic function of LCOR in HR^+^ BC, we used a computational regression interaction model between LCOR and the different nuclear receptors affecting the APM signatures and the cytolytic score defined by the granzyme and perforin index (51) in TCGA HR^+^ BC clinical samples. This computation analysis showed 3 main nuclear receptors affecting the correlation of LCOR with the cytolytic score and APM functions (Figure 3D): *ESR1*, *ESR2*, and *NR2F2*. The last is also part of the ERα complex. Therefore, this clinical analysis suggests that LCOR-mediated immunogenicity is mostly constrained by ERα in HR^+^ BC, which aligns with our preclinical data.

### Disruption of LCOR-ERα interaction restores LCOR immunogenic activity in HR^+^ BC.

**Figure 4:**
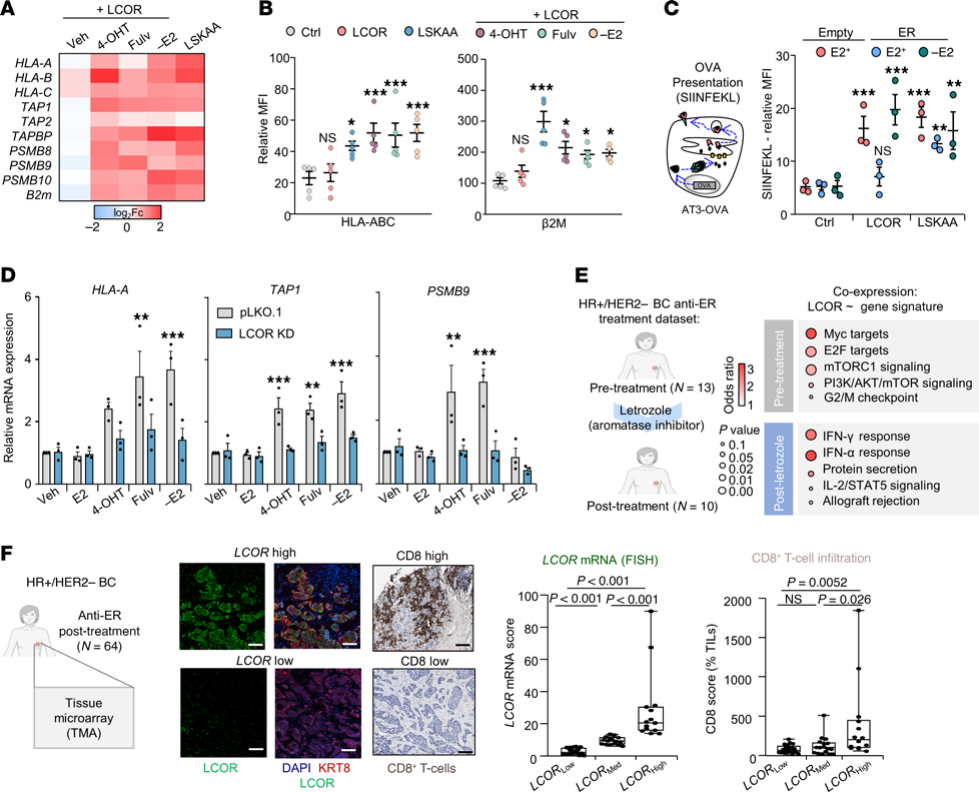
Molecular disruption of the LCOR-ERα interaction restores LCOR immunogenic activity in HR^+^ BC. (**A**) RT-qPCR analysis of MHC-I in MCF-7 cells treated with the indicated anti-ER drugs. Data are represented as log_2_(fold change) of the normalized expression of the vector control condition. *n* = 3 individual biological replicates; data represents the mean. (**B**) Flow cytometric analysis of HLA-ABC and β2m levels in MCF-7 cells under the same conditions in **A**. *n* = 5 independent biological replicates. Data represent the MFI relative to the isotype control ± SEM. (**C**) Flow cytometric analysis of the OVA peptide SIINFEKL presented by H2-K1^b^ in AT3-OVA vector control (Empty) or ER-OE (AT3-ER) cells in the different conditions: control, LCOR-OE, and LSKAA-OE cells treated with regular (E2^+^) or E2-depleted media (–E2, charcoal-stripped FBS). *n* = 3 independent biological replicates; data represent the MFI relative to the isotype control ± SEM. (**D**) RT-qPCR analysis of *HLA-A*, *TAP1*, and *PSMB9* genes in vector control (pLKO.1) and LCOR-KD MCF-7 cells treated with vehicle or the respective anti-ER drugs (1 μM) or 10% charcoal medium for 24 hours. *n* = 3 independent biological replicates. Data show the mean ± SEM. (**E**) Pathway analysis of LCOR-coexpressed transcripts for pre- and post-letrozole therapy groups of patients with BC (46). Pathways are ranked on the basis of the OR and *P* value. (**F**) ISH images of specimens from patients with BC. ISH stratification of *LCOR*^hi^ and *LCOR*^lo^ HR^+^ tumors treated with endocrine therapy (aromatase inhibitor letrozole). The tumor fraction was detected by DAPI and KRT8 staining. Scale bars: 200 μm. IHC staining of the corresponding high and low CD8^+^ T cell–infiltrated tumors. TMA samples were stratified by *LCOR* mRNA levels into low, medium, and high expression of *LCOR* from ISH staining and the corresponding CD8^+^ T cell tumor infiltration score. *P < 0.05, **P < 0.01, and ***P < 0.001, by 1-way ANOVA (**F**) and 2-way ANOVA (**B**–**D**. Cells were gated from P3 (Supplemental Figure 8A) in **B** and **C**.

Next, we hypothesized that the molecular inhibition of ERα could restore the immunogenic effects of LCOR. First, we used different ERα modulators (4OH tamoxifen, fulvestrant, E2 supplementation, and E2-depleted media) to characterize the LCOR-ERα interaction after in vitro treatment using a proximity ligation assay (PLA). As expected, MCF-7 LCOR-OE cells showed increased numbers of interactions with E2 supplementation and a significant decrease with anti-estrogen treatment (Supplemental Figure 4A). Remarkably, our LSKAA construct (LCOR mutant without a NR-binding domain) exhibited the lowest number of interactions (Supplemental Figure 4A). Subsequently, using the same molecular inhibitors of ERα, we could restore the immunogenic function of LCOR in HR^+^ BC by increasing APM expression in MCF-7-LCOR–OE cells measured by flow cytometry and quantitative reverse transcription PCR (RT-qPCR) (Figure 4, A and B). Accordingly, LSKAA also increased the APM pathway (Figure 4, A and B).

To validate the antigen presentation ability of LCOR in the ER^+^ context, we checked the OVA peptide SIINFEKL Mhc-I presentation in AT3 murine cells. Both LCOR and LSKAA increased OVA presentation in AT3-OVA cells in the absence of ERα. However, in AT3-ER cells, only LSKAA augmented the presentation under regular E2 conditions, and E2 depletion from media was necessary to increase SIINFEKL presentation in LCOR-OE cells (Figure 4C), which was also reflected by APM gene expression measured by qRT-PCR (Supplemental Figure 4B). Finally, to demonstrate the requirement of LCOR for APM expression upon anti-ERα strategies, we treated LCOR-knockdown (LCOR-KD) MCF-7 cells with anti-ERα and observed increased *HLA-A*, *TAP1*, and *PSMB9* mRNA expression in control cells, but not in LCOR-KD cells (Figure 4D and Supplemental Figure 4C), suggesting that the anti-estrogen therapies can increase APM depending on LCOR activity (Figure 4D). Overall, these results demonstrate that the molecular therapeutic intervention targeting LCOR-ERα interaction restores LCOR immunomodulatory functions in HR^+^ BC.

To determine in patients whether endocrine therapies inhibiting ERα drive similar LCOR effects, we checked pathways gene coexpression with LCOR in pre- and post-letrozole RNA-Seq cohorts (43). As expected, only post-treated (after ERα inhibition) samples showed LCOR coexpression with immunogenic pathway genes, such as IFN-α and IFN-γ responses (Figure 4E). Additionally, to further support these data, we used a tissue microarray (TMA) of 64 tumors from patients with HR^+^ BC who were treated with endocrine therapy (aromatase inhibitors) (52). We performed *LCOR* ISH and anti-CD8 IHC (Figure 4F) to evaluate the levels of CD8^+^ T cell infiltration in patients with low, medium, and high *LCOR* expression in tumors (Figure 4F). In alignment with our preclinical and clinical data, after endocrine therapy, *LCOR*^hi^ tumors had higher numbers of infiltrating CD8^+^ T cells than did *LCOR*^lo^ tumors (Figure 4F), suggesting that the disruption of ERα in patients’ samples allowed LCOR to turn on its immunogenic functions. Overall, these results demonstrate that the disruption of LCOR from ERα increased APM expression and the immunogenic properties of HR^+^ BC.

### ERα disruption relocates LCOR chromatin binding and increases LCOR location in APM loci.

**Figure 5:**
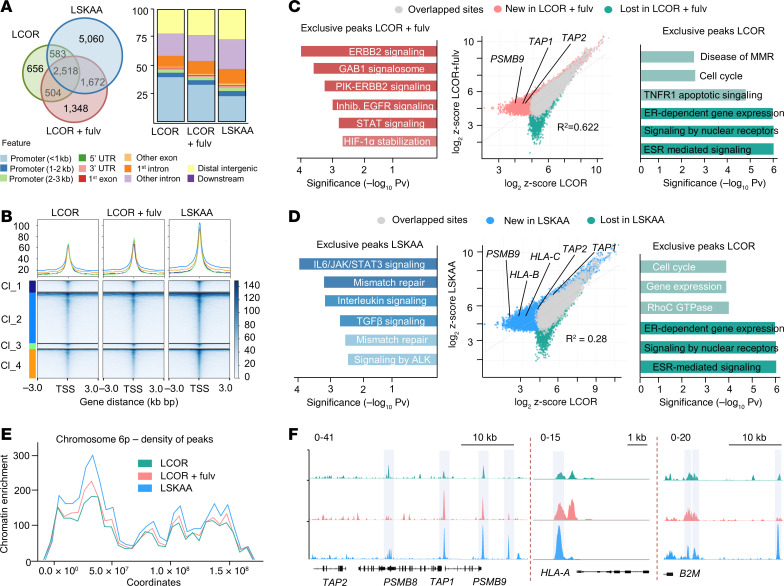
ER disruption relocates LCOR chromatin binding and increases LCOR location in APM loci. (**A**) Venn diagram of overlapping peaks across conditions from MCF-7 ChIP-Seq: LCOR, LCOR+Fulv (1 μM, 24 hours), and LCOR_LSKAA. Bar plot depicts genomic features and gene-regulatory element distribution of ChIP-Seq samples in the indicated conditions. (**B**) Tornado plot of ChIP-Seq *k*-means clustering based on the *z* score from the 3 different conditions with 4 representative clusters (Cl). (**C** and **D**) Comparative analysis and scatter plot showing genome-wide changes of LCOR binding in LCOR-untreated versus LCOR+Fulv-treated (**C**) or LSKAA (**D**) MCF-7 cells. Red dots denote sites induced by fulvestrant, green dots denote sites lost by fulvestrant, blue dots denote sites induced by LSKAA, and gray dots denote sites unchanged by treatment. The *R^2^* value is shown. Analysis of the REACTOME pathway of the gained and lost peaks ranked by –log_10_ *P* value. (**E**) Genomic distribution of LCOR in the chromosome 6p locus (MHC-I/APM region). Signal was obtained from the average of the ChIP-Seq analysis of 2 independent biological replicates. (**F**) ChIP-Seq peak occupancy of LCOR in genes involved in the APM pathway. The occupancy and scale are indicated. Shadows denote comparative peak enrichment. Data show a biological replicate.

To understand how ERα influences LCOR function and chromatin binding, we performed ChIP-Seq in human MCF-7-LCOR–OE cells treated with vehicle, anti-estrogen (fulvestrant), or LCOR_LSKAA. Of the total 12,341 peaks, we found that 2,518 (20.4%) were shared across conditions; 1,348 (10.9%) were gained with LCOR plus fulvestrant (LCOR+Fulv); and 5,060 (41%) were de novo gained peaks exclusive of LSKAA located at distal regulatory regions (Figure 5A and Supplemental Figure 5, A and B). *K*-means clustering analysis grouped all the dynamic peaks across conditions in 4 different clusters that were interrogated through Homer analysis (53) (Figure 5B and Supplemental Figure 5C). Homer analysis showed that most of the clusters were related to AP-1 motifs: cluster 1 of LCOR and LSKAA co-shared regions and cluster 2 of LSKAA private motifs. Cluster 3 was very small, with exclusive peaks in the LCOR+Fulv condition. Interestingly, cluster 4 comprised new regions gained for both LSKAA and LCOR+Fulv, which showed an increase in motifs related to inflammatory factors such as ATF3, NFY, and NF-κB/p65 (Supplemental Figure 5C).

To gain deeper insight into LCOR de novo regulatory functions, we analyzed the differentially regulated peaks across conditions (Figure 5, C and D). Fulvestrant treatment made LCOR gain access mainly to cell-cycle–related genes but also to STAT-related signaling, which is associated with IFN and APM signaling (54). Conversely, LCOR peaks lost by fulvestrant treatment were enriched in ER and nuclear receptor signaling (Figure 5C). LSKAA also presented stronger enrichment in STAT and IL signaling compared with LCOR, with a reduced association in ER-mediated signaling (Figure 5D). *R^2^* linear regression of peaks between conditions showed a lower value between LSKAA and LCOR than between LCOR+Fulv and LCOR, again indicating a greater molecular relocalization of LSKAA than LCOR+Fulv. LCOR+Fulv and LSKAA shared peaks are related to STAT signaling (Supplemental Figure 5D), with no additive effect of LSKAA+Fulv binding to APM motifs (Supplemental Figure 5E), indicating that LSKAA, by avoiding ERα interaction, already showed full freedom to induce APM.

Next, we evaluated LCOR association with APM-regulatory loci. Fulvestrant treatment and LSKAA exhibited a higher enrichment in the short arm of human chromosome 6, where most of the APM genes are located (Figure 5E), including *TAP2, PSMB9*, *TAP1*, *PSMB8*, *HLA-A*, and *B2M* (Figure 5F). We validated our results through ChIP-qPCR assays of APM loci including different anti-ERα compounds: 4-OH tamoxifen, fulvestrant, and E2 depletion from media. Accordingly, all treatments increased LCOR binding to APM genes to an extent similar to that seen with LSKAA (Supplemental Figure 5F). Overall, these results demonstrate that by interfering with the LCOR-ERα interaction, LCOR’s chromatin binding was relocated to de novo immunomodulatory gene–regulatory regions. Therefore, by molecular targeting of the ERα-LCOR interaction, we were able to reconfigure LCOR function based on the LCOR switching effect.

### LSKAA turns immune-cold HR^+^ tumors into immune-hot tumors.

**Figure 6:**
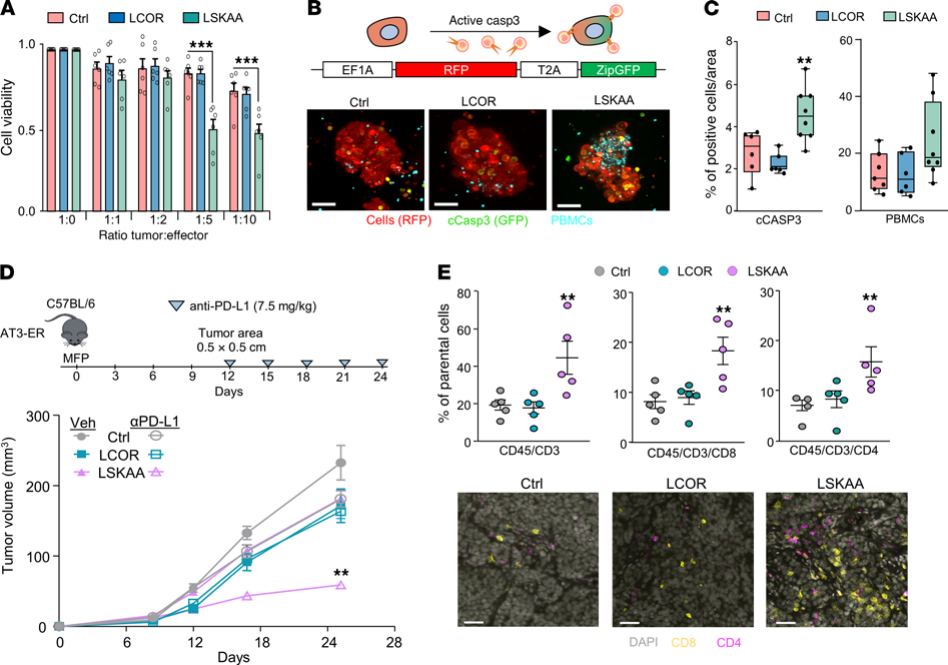
LCOR-LSKAA turns immune-cold HR^+^ tumors into immune-hot tumors. (**A**) CTL assay of MCF-7 cells after 48 hours of coculturing with PBMCs at different ratios of effector (E) and tumor (T) cells in control, LCOR-OE, and LSKAA-OE conditions. (**B**) Confocal microscopy images of MCF-7-ZipGFP, control, LCOR-OE, and LSKAA-OE cells cocultured with PBMCs. Tumor cells are labeled with mCherry (red), PBMCs with Cell Tracker Deep Red (cyan), and apoptotic cells with ZipGFP (green). Scale bars: 10 μm. (**C**) Quantification of caspase 3^+^ tumor cells and PBMC infiltration per tumoroid area. Box plots represent the IQR with individual data points. (**D**) Growth curves of orthotopically implanted AT3-ER tumors transduced with backbone (control), LCOR-OE, or LSKAA-OE plasmids. Mice were treated with vehicle or anti–PD-L1 antibody at the indicated doses and regimes. *n* = 10 tumors for each condition. (**E**) Flow cytometric analysis of the percentage of tumor-infiltrated lymphocytes defined as CD45^+^CD3^+^, CD45^+^CD3^+^CD8^+^, or CD45^+^CD3^+^CD4^+^ T cells in anti–PD-L1–treated, control (backbone), LCOR-OE, and LSKAA-OE tumors. *n* = 5 individual biological replicates. Tumor tissue IF analysis of CD4^+^ and CD8^+^ T cells from control, LCOR-OE and LSKAA-OE AT3 tumors. Scale bars: 50 μm. Data represent the mean ± SEM (**A**, **D**, and **E**). **P < 0.01 and ***P < 0.001, by 2-way ANOVA (**A**, **C** and **E**) and mixed-design ANOVA (**D**). Cells in **E** were gated as shown in Supplemental Figure 8B. Casp3, caspase 3; cCASP3, cleaved caspase 3.

By manipulating ERα interaction with LCOR, we tested the effects of LCOR-mediated APM induction on HR^+^ tumor immunity. We performed in vitro coculturing of MCF-7 cells expressing LCOR or LSKAA with human PBMCs. Consistent with the mechanism described above, MCF-7-LCOR–OE cell viability was not reduced compared with the control condition, however the LSKAA-OE cells resulted in a greater cellular killing mediated by the immune cells (Figure 6A). To confirm the T cell–mediated killing, we measured cell death by necrosis (7-ADD) and apoptosis (annexin V), and only LSKAA-OE cells showed significantly increased immune-mediated tumor cell killing compared with MCF-7 control cells (Supplemental Figure 6, A–C). Additionally, we generated MCF-7 tumor 3D spheroids transduced with ZipGFP plasmid (labels tumor cells in RFP and caspase 3 activity in GFP) as a readout of apoptosis tumor cell death. Using this 3D system, we could show how LSKAA-OE cells enhanced PBMC infiltration and caspase 3 (ZipGFP^+^) activity, indicating immune-mediated tumor apoptosis (Figure 6, B and C, and Supplemental Figure 6D). To validate our results using specific antigen recognition systems, we used the OVA/OT-I system in AT3-ER-OVA cells. Consistently LCOR did not increase T cell killing of AT3-OVA-ER tumor cells, but LSKAA or LCOR with E2-depleted media did increase OT-I CD8^+^ T cell–mediated killing (Supplemental Figure 6E). These results functionally validate the previous mechanistic observations showcasing the potential molecular targeting of LCOR-ERα interaction in HR^+^ BC.

In order to test our molecular strategy in preclinical IC settings in vivo, we next performed MFP injection of AT3-ER cells with LCOR or LSKAA into IC C57BL/6 mice. As expected, anti–PD-L1 therapy had little effect on tumor growth in control cells or LCOR-OE cells, but we observed strong tumor suppression in LSKAA-OE tumors (Figure 6D), along with increasing immune cell infiltration, especially of CD8^+^ T cells (Figure 6E). Therefore, LSKAA by preventing ERα interaction, mediated LCOR immunogenic functions, converting these ER^+^ BC cold tumors into hot tumors and facilitating the immunotherapeutic response to anti–PD-L1.

### Leveraging LSKAA for immunotherapeutic response in human-derived HR^+^ models.

**Figure 7:**
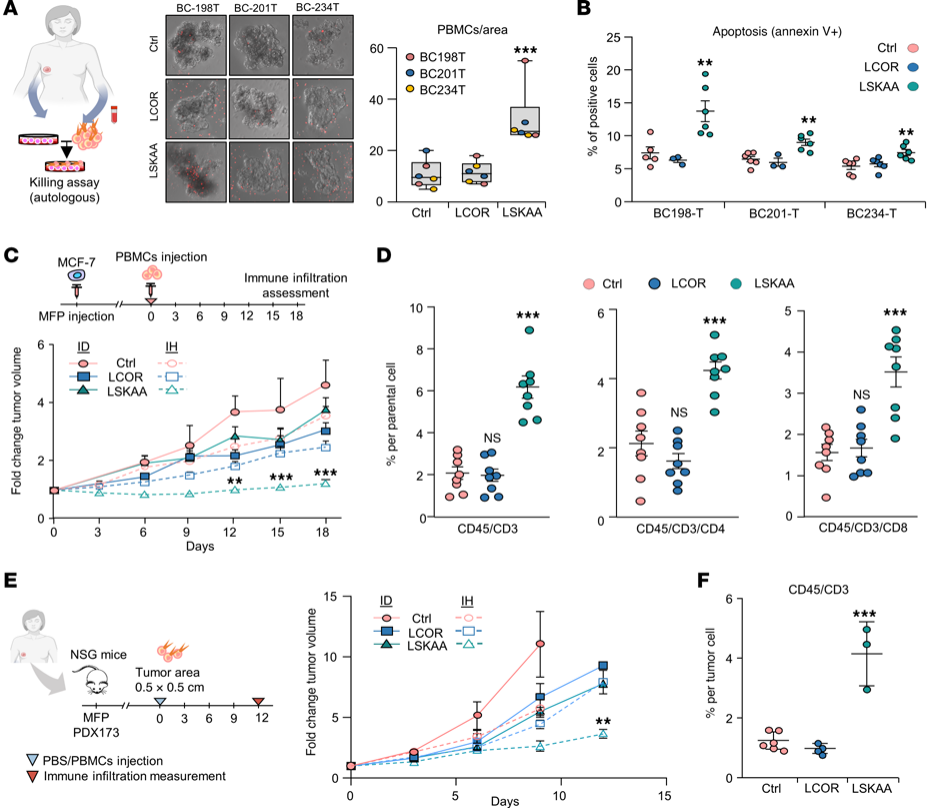
LSKAA-mediated immune reaction in different human HR^+^ BC specimens. (**A**) Confocal microscopy images of control, LCOR-OE, and LSKAA-OE PDOs from 3 patients with HR^+^ BC cocultured with autologous PBMCs. PBMCs are labeled with Cell Tracker (red). Original magnification, ×20. Graph shows quantification of PBMC infiltration across conditions. (**B**) Flow cytometric analysis of cell death: apoptosis (annexin V) from control, LCOR-OE and LSKAA-OE PDOs cocultured with autologous PBMCs. *n* = 5 independent biological replicates. (**C**) Growth curves of orthotopically transplanted tumors in NSG mice with (IH) or without (ID) injection of 10 × 10^6^ PBMCs. *n* = 8 mice for each condition. (**D**) Flow cytometric analysis of the percentage of tumor-infiltrated lymphoid cells defined as CD45^+^CD3^+^, CD45^+^CD3^+^CD8^+^, and CD45^+^CD3^+^CD4^+^ T cells in control, LCOR-OE, and LSKAA-OE MCF-7 tumors. *n* = 8 individual biological replicates. (**E**) Growth curves of orthotopically implanted HR^+^ PDX173 in NSG mice under the following conditions: control, LCOR-OE, and LSKAA-OE plasmids. Mice were randomized into vehicle (ID) or injection of 10 × 10^6^ PBMCs (IH) groups. *n* = 5 mice. (**F**) Flow cytometric analysis of the percentage of TILs (CD45^+^CD3^+^) in PDX173 control, LCOR-OE and LSKAA-OE cells. *n* = 5 mice for control and LCOR; *n* = 3 mice for LSKAA. Data represent the mean ± SEM (**B**–**F**). **P < 0.01 and ***P < 0.001, by 1-way ANOVA (**A**), 2-way ANOVA (**B**, **D**, and **F**), and mixed design ANOVA (**C** and **E**). Cells were gated as shown in Supplemental Figure 8A in **B** and as shown in Supplemental Figure 8B in **D** and **F**.

In order to extend our findings to a clinically relevant test, we investigated the immunogenic role of LSKAA in different patients using patient-derived organoid models. Hence, we generated patient-derived organoids (PDOs) from HR^+^ BC samples and cultured them in 3D Matrigel. We established several specimens with different clinical pathological features (Supplemental Figure 7A). We confirmed that ER^+^ PDOs retained their expression of the luminal marker KRT8 and ERα (Supplemental Figure 7B). In parallel, we collected blood samples from the same BC patients to isolate autologous PBMCs. PDOs were transduced with either control, LCOR, and LSKAA overexpression vectors. These PDOs were then cultured in nonadherent conditions to allow for spheroid formation and cocultured with autologous PBMCs for each PDO (Figure 7A). As observed with HR^+^ BC cell lines, LSKAA-transduced PDOs had a higher number of infiltrating PBMCs compared with control and LCOR-transduced PDOs (Figure 7A). LSKAA expression in PDOs significantly increased the immune killing compared with control PDOs, as measured by markers of apoptosis (annexin V) and necrosis (7-ADD) (Figure 7B and Supplemental Figure 7C), as well as increased APM (Supplemental Figure 7D). Therefore, LSKAA immune effects on PDOs confirmed the utility of targeting LCOR-ERα interactions to elicit immune responses across different HR^+^ BC patient avatars using immune-autologous settings.

Using preclinical IH models, we injected MCF-7 control cells, LCOR-OE cells, or LSKAA-OE cells into the MFP of immune-compromised NSG mice and administered 10 × 10^6^ PBMCs through the tail vein. LSKAA-OE cells significantly decreased tumor growth compared with control or LCOR-OE cells (Figure 7C). At the end of the experiment, all 3 conditions had similar levels of circulating PBMCs in the blood of the mice (Supplemental Figure 7E). Flow cytometric analysis and tissue IF of harvested tumors showed that LSKAA tumors had higher infiltration of CD4^+^ and CD8^+^ T cells compared with control and LCOR tumors (Figure 7D and Supplemental Figure 7F).

Finally, we used an HR^+^ patient–derived xenograft (PDX). PDX173 showed consistent growth after several passages and retained ERα expression (Supplemental Figure 7G). PDX173 cells were isolated and infected with control, LCOR-OE, or LSKAA-OE vectors and implanted into NSG mice via the MFP. When tumors reached 0.5 × 0.5 cm^2^ in size, a group of mice were adoptively transferred with PBMCs to test humanized immune activity in preclinical settings in vivo (Figure 7E). As expected, based our previous findings (37), LCOR-OE and LSKAA-OE PDXs showed a minor degree of tumor-intrinsic activity in NSG ID mice. However, in mice adoptively transferred with PBMCs, LSKAA-OE PDXs had a greater reduction in tumor growth compared with control or LCOR-OE PDXs (Figure 7E), along with a higher percentage of immune infiltration, measured as the fraction of CD45^+^CD3^+^ immune cells (Figure 7F). Overall, these human preclinical assays demonstrate that disruption of LCOR and ERα interactions reconfigured HR^+^ tumor immunity toward high immune reactivity and posit the molecular targeting of ERα-LCOR interactions as a potential immune-based therapy in HR^+^ BC tumors.

## Discussion

Our study unveils why HR^+^ BCs are immune-cold tumors that are poorly infiltrated and poorly responsive to immunotherapy (9, 22) and present a significant challenge in BC immune oncology. We reveal mechanistic implications of ERα signaling itself as a driver of immunosuppression and immunotherapy resistance, in part by preventing LCOR from executing the immunogenic APM program. Therefore, this study offers conceptual advances in the understanding of ERα biology in BC immunity and proposes molecular targeting of LCOR-ERα interaction to overcome immune evasion and immunotherapy resistance. These findings are timely, with recent clinical observations (8, 10, 12) showing how within the HR^+^ BC subtype, ERα expression is associated with a reduced immune-reactive signature (10), immune infiltration (20, 21, 23), and responses to ICB therapies. In addition, our study demonstrated that the molecular targeting of the LCOR-ERα interaction is an effective immune-based complementary therapy to overcome this unmet clinical need in HR^+^ BC.

Recent publications using transcriptomics signatures have associated the negative correlation of ERα and tumor immunity to a basal-like phenotype of ER^lo^HR^+^ BC tumors (10, 11, 28, 55, 56). Our results further suggest that ERα is a main driver of immunosuppression associated with the luminal phenotype. In agreement with our observations, recent mechanistic studies have reported ERα suppression of type I and II IFN signaling interferes with STAT transcription factors (57, 58) and is reverted through the application of anti-ER therapies in vitro (59). Additional innate immunity ER-immunosuppressive effects have also been shown (59–62) and may act in consonance with the ERα restriction of LCOR tumor–mediated effects on the adaptive immune system described here. Still, the mechanistic understanding and functional consequences of ERα in tumor immunology in BC have yet to be thoroughly characterized and are thus still poorly understood. In part, the lack of good ER^+^ BC murine models to use in IC settings in vivo has limited preclinical studies on HR^+^ BC immunology. In order to overcome this limitation and focus on ER biology, we took a simplistic and direct approach by ectopically introducing ERα (*ESR1* gene) into a mouse BC cell line. The sole introduction of ERα was sufficient to induce immunosuppression and reduce the immunotherapeutic response. In addition, complementary models using human cells challenged with PBMCs also confirmed that ERα expression — by driving immunosuppression — is positively selected under immune pressure (63). Our MCF-7 preclinical model showed elevated levels of *ESR1* and *FOXA1* mRNA, which are genes that have also been found in nonresponder patients in immunotherapy trials (11, 12). These findings have important implications for ER^+^ tumor immunology and treatment of HR^+^ BCs.

LCOR was first described as an agonist-activated NR corepressor with growth suppressor functions. However, we recently described LCOR as a potent inducer of immunogenic genes potentiating ICB responses in preclinical models of TNBC (37, 38). Here, we unexpectedly found that LCOR can act as an immunogenic instigator in the presence of ERα being hijacked and participating in its signaling, what results in poor immunogenicity of HR^+^ tumors. This bivalent transcriptional activity has already been described for other NR corepressors, namely NCOR (64) and RIP140 (65), illustrating the complexity of this family of proteins. Indeed, different types of NRs are expressed in other cancer types with reported influence on tumor immunology (66–68). Therefore, these effects of ERα on tumor cells are aligned with the biology of other NRs and also with the antiinflammatory effects of steroids on immune cells. Although not explored in this study, we envision that this LCOR mechanism is likely to be general across these cancer types. Therefore, further LCOR research and NR modulation strategies should be performed in other hormone-depended cancer types, such as prostate cancer.

In the absence or presence of ERα, we found different genomic localizations of LCOR. By preventing ERα activity, LCOR shifts from ERα-binding sites to APM- and IFN-mediated gene-binding sites in the genome, thus constituting an LCOR switch. This switch reflects a reconfiguration of tumor immunogenic properties upon ERα targeting with conventional endocrine therapies. Blocking ERα activity leads to a stoichiometric advantage of LCOR over ERα that would explain the capacity to induce APM. Finally, introduction of the LSKAA construct that cannot interact with NRs can directly activate the transcription of APM at a higher magnitude than anti-ERα compounds can achieve. Therefore, the use of LSKAA-encoding mRNA synthetic therapy with nanoparticles should be considered. Indeed, we have intellectual property (PCT/EP2024/056727) to exploit this technology for clinical applications, which we believe could be highly relevant for the treatment of advanced HR^+^ BC.

Patients with ERα positivity of at least greater than 1% are considered to have ER^+^ BC, with 10%–50% of these patients classified as having ER^lo^ BC (69). Recent reports have argued the necessity to redefine the molecularly intrinsic BC subtypes according to the expression pattern (MammaPrint or PAM50) to better allocate patients in the newly available treatment options (26, 70, 71). Our study also has immediate clinical implications, as it confirms the need to stratify patients with HR^+^ BC on the basis of their ER levels, in addition to LCOR levels, as biomarkers for optimized clinical benefit of immune-based therapies. We showed how endocrine therapies in HR^+^ BC increased immune-related signatures and immune infiltration depending on LCOR levels, which was also confirmed in our functional models. These results provide a rationale for the use of endocrine therapies concomitantly with immunotherapy and include not only stratification in the neoadjuvant setting, but also potential longitudinal assessment of ER^+^ with disease progression after advanced lines of treatment. Clinical trials evaluating responses to this combination showed elevated infiltration of reactive T cells within tumors and increased T cell reactivity (11, 72, 73). Still, results do not show a clear benefit, thus encouraging new clinical trial designs testing this combination while keeping in mind that the benefit still depends on LCOR levels in patients. Importantly, this study offers a readily feasible strategy of molecular targeting of the LCOR/ERα axis using endocrine therapy and highlights potential future approaches leveraging LSKAA-encoding therapies to convert cold tumors into hot tumors, which is the overarching challenge in HR^+^ BC immunity and immunotherapy in these patients.

## Methods

### Sex as a biological variable.

Our study exclusively included female mice because BC mostly occurs in female individuals. Fresh patient material and clinical datasets included only female patients with BCs. It is unknown whether the findings are relevant for male BC.

### Animal studies.

C57BL/6J, C57BL/6-Tg (TcraTcrb)1100Mjb/J (OT-I) and NOD.Cg-Prkdc^scid^Il2rg^tm1Wjl^/Szj (NSG) mouse strains were obtained from Charles River Laboratories. For orthotopic models, 8- to 10-week-old female mice were randomized into different experimental groups, and tumor cells were implanted into the MFP in 1:1 PBS/Matrigel. For tumor growth studies, 1 × 10^6^ MCF-7, 0.5 × 10^6^ AT3 and 1 × 10^6^ PDX173 cells were injected into NSG or C57BL/6J mice. Mice were supplemented with E2 (17β-estradiol, Merck E8875) at 0.4 μg/mL in the drinking water unless otherwise stated in the figure legend. Tumor-bearing NSG mice were randomized and injected with 10 × 10^6^ PBMCs through tail vein injection, when primary tumors reached 0.5 × 0.5 cm^2^ tumor area. Tumor volume was measured twice a week with digital caliper (Merck, Z503576-1EA) and calculated as follows: π × length × width^2^/6. 7.5 mg/kg anti–PD-L1 (BioXCell, clone 10F.9G2, catalog BE0101), or PBS was applied twice a week starting at the 0.5 × 0.5 cm^2^ tumor area. Mice were euthanized once tumors reached 1,000 mm^3^ in size or the animal’s health was compromised.

### PDOs and PDXs.

Patient-derived tumor specimens were obtained from surgical resections of female patients with HR^+^ BC at the Hospital del Mar (Barcelona, Spain). Tumors were processed and cultured as previously described (74, 75). Additional components were added to PDOs culture media to sustain growth and ERα expression: 10 ng/mL amphiregulin (Biotechne, 262-AR), 10 μg/mL insulin (MilliporeSigma, 91077C) and 1 μg/mL 4-OH Progesterone (MilliporeSigma, P8783). PDX173 (ER^+^/HER2^+^) was obtained from the Joaquín Arribas laboratory at the Vall d’Hebron Institute of Oncology. Sections (0.1 × 0.1 cm^2^) were transplanted s.c. onto contralateral mammary glands in NSG mice supplemented with E2 (0.4 μg/mL beverage).

### Cell lines, culture conditions, and treatments.

MCF-7, MDA-MB-231 and HEK293T cell lines were obtained from Y. Kang Lab at Princeton University and cultured according to the American Type Culture Collection (ATCC). AT3 cells were obtained from ATCC. Cells were validated and routinely checked for Mycoplasma infection. 17β-Estradiol (Merck, 50-28-2) was used at 10 ng/mL; 4-OH Tamoxifen (Merck, 68047-06-3) and fulvestrant (Merck, I4409) at 10 μg/mL; doxycycline (Merck, D5207) at 1 μg/mL; and charcoal-stripped FBS (Thermo Fisher Scientific, A3382101) at 10% in regular media. PBMCs were isolated and cultured as previously described (76).

### Isolation of tumor-infiltrating immune cells and flow cytometric analysis.

Harvested tumors were mechanically digested followed by 30 minutes of enzymatic digestion with 2 mg/mL collagenase A and 0.5 mg/mL DNAse I. Cells were strained through a 100 μm strainer and incubated for 5 minutes at 37ºC in ACK lysing buffer. Cells were strained through a 40 μm strainer and then counted and processed for flow cytometric staining and analysis. For mouse blood samples, 50 μL blood was extracted through chin puncture. Blood was incubated in 2 mL ACK lysis buffer for 2 minutes, and pellet cells were processed for flow cytometric staining. For IFN-γ staining, single-cell suspensions were stimulated with the stimulation cocktail containing phorbol-12-myristat-13-acetate plus ionomycin and brefeldin A (BioLegend, 423304) for 4 hours at 37°C. Cells were incubated with FcγIII/II receptor CD16/CD32 (BioLegend, 101302) and True-Stain Monocyte blocking antibodies (BioLegend, 426102) for 10 minutes at 4°C, followed by surface staining with antibodies for 20 minutes at 4°C. For intracellular staining, fixable viability dye live/dead blue (Invitrogen, Thermo Fisher Scientific, L23105) was used to stain dead cells. Cells were fixed and permeabilized with the Foxp3/Transcription Factor kit (Invitrogen, Thermo Fisher Scientific, 005-52300) according to the manufacturer’s protocol, followed by a 1-hour incubation at 4°C with intracellular antibodies.

### CTL assay.

For mouse CTL assays, T cells were isolated from OT-I mouse splenocytes using CD8a^+^ T cell isolation kit (Miltenyi Biotec, 130-104-075). AT3-OVA cells (*n* = 5,000 cells) were seeded in 24-well plates with OT-1^+^ T cells in 10% FBS RPMI media at different ratios. Cells were treated with 1 μg/mL mouse IFN-γ (Gibco, Thermo Fisher Scientific, PMC4031) 24 hours prior to coculturing for control and ER experiments. After 96 hours, cell viability was assayed with 0.25% crystal violet staining (Merck, HT90132). Data are represented as cell viability relative to the non-coculture condition. For immune phenotyping, OT-1 T cells were collected after 48 hours of coculturing. For human CTL assays, 75,000 MCF-7 cells were plated in 24-well plates with 10% FBS RPMI media. PBMCs were added at different ratios. After 48 hours, media were aspirated, and attached MCF-7 cells were processed for crystal violet or cell death marker assessment by FACS. For spheroid assays, 5,000 MCF-7 or PDOs cells were seeded in low-adherence, 96-well plates supplemented with 10% FBS RPMI. After 48 hours, spheroids were collected and cocultured with 20,000 PBMCs from healthy donors or patients (autologous PBMCs). After 48 hours, samples were imaged and processed for death markers through flow cytometry.

### IF, confocal, and IHC analysis.

For IF, cells were seeded in coverslips, fixed in 4% PFA, and permeabilized with methanol. Samples were blocked (2.5% BSA, 0.25% Triton-100 in PBS) and incubated o/n at 4ºC with primary antibodies (see Supplemental Methods and Supplemental Table 1). After secondary antibody incubation, cells were mounted with DAPI mounting solution (SouthernBiotech, 0100-20). Images were acquired using an upright Nikon Eclipse Ni-E fluorescence microscope (Nikon).

Living spheroids cocultured with PBMCs were imaged with an SP8 Leica confocal microscope system. PBMCs were labeled with cell trackers (green CMFDA C7025 or CMPTX deep red 11514267, Thermo Fisher Scientific). Samples were imaged using a *Z*-stack of 2 μm. Images were projected to obtain a maximum projection of spheroids.

For IHC or immunohistofluorescence (IHF), paraffin-embedded tissue samples were cut into 3 μm slices. After the tissues were dried and deparaffinized, antigens were retrieved with a 0.5 M citrate pH 6 pressure bath for 20 minutes. Tissue slices were blocked and incubated o/n at 4ºC with primary antibodies. For IF tissues, fluorescence-conjugated antibodies were incubated for 1 hour at room temperature and then washed and mounted in DAPI mounting solution. For IHC, HRP-conjugated secondary antibodies were added and washed, and signal was revealed using the ImmPress IgG polymer peroxidase kit (MP-7452, VectorLabs) by the DAB method. Samples were stained with hematoxylin and dehydrated for mounting. Images were processed and quantified with Fiji software.

### Viral production and transduction of cell lines.

HEK293T cells were transfected with lentiviral plasmids together with pocket vesicular stomatitis virus G glycoprotein (VSV-G) and gag-pol plasmid pCMV-R8.91, following the lentiviral packaging protocol (77). HEK293T supernatants were collected and filtered 48 and 72 hours after transfection. Cells were transduced with virus-conditioned media with 8 μg/mL polybrene and selected with the corresponding antibiotic resistance.

### ChIP and ChIP-Seq library preparation.

For ChIP-qPCR and sequencing, cells were grown in 150 mm^2^ plates, crosslinked for 10 minutes in 2% PFA, and lysed. Cells were sonicated in ten 30-second cycles ON/OFF using Bioruptor Pico Tubes (Diagenode) with sonication beads (Diagenode) in a Bioruptor sonicator. Sonicated chromatin was incubated with anti-HA antibody at 4ºC in rotation o/n. Chromatin-antibody complexes were pulled down using Protein G dynabeads (10009D, ThermoFisher). Isolated chromatin was de-crosslinked at 65ºC for 4 hours and DNA was purified with the Qiagen DNA purification kit (Qiagen, 56304). All primers for ChIP-qPCR used in the study are listed in Supplemental Table 3. For ChIP-Seq, chromatin quality and quantity were checked using the Agilent Bioanalyzer. Libraries were sequenced as 50 bp paired end on the HiSeq 2500 platform (Illumina). The ChIP-Seq analysis methodology is described in Supplemental Methods.

### Molecular cloning and plasmids.

The pLEX overexpression plasmid was obtained from the Y. Kang Laboratory (Princeton University, Princeton, New Jersey, USA). Backbone_HA, *LCOR*_HA, *LSKAA*_HA, *Lcor*, and *Lskaa* were subcloned into the pLEX plasmid using SpeI and AgeI enzymes (New England Biolabs). OVA peptide was subcloned into the pLEX plasmid (pLEX-OVA). pHAGE-ESR1 was obtained from Addgene (plasmid no. 116737). For the generation of an inducible system of LCOR expression, the PB-TRE-dCas9-VPR plasmid, obtained from Addgene (plasmid no. 63800), was digested with AgeI and NehI enzymes to release dCas9-VPR. *LCOR* and *LSKAA* were amplified from the pLEX plasmid and subcloned using the same restriction enzymes. The ZipGFP plasmid was purchased from Addgene (plasmid no. 81241) and subcloned into the lentiviral overexpression system by VectorBuilder. For gene-KD assays, shRNAs targeting the human *LCOR* gene were purchased from Merck (TRCN0000016306). All plasmids used in this study are listed in Supplemental Table 2.

### Flow cytometric analysis and cell sorting.

Cells were collected and resuspended in FACS buffer (PBS 2% FBS and 2 mM EDTA) and were stained according to the manufacturer’s parameters (see Supplemental Table 1). Flow cytometric data were collected on a Fortessa Flow cytometer (BD) using FACSDiva 9.0 (BD) software or an Aurora 5L (Cytek) instrument. Data were analyzed using FlowJo 10.8.1 and normalized to the corresponding isotype control. For cell sorting, FACSAria (BD) equipment was used.

### RNA isolation and RT-qPCR analysis.

Total mRNA was purified using the RNeasy Mini Kit (Qiagen, 74104). Purified mRNA (1 μg) was reversed transcribed into cDNA using the High-Capacity cDNA Reverse Transcription Kit (Life Technologies, Thermo Fisher Scientific, 4368814). qPCR was performed using LightCycler 480 SYBR Green I Master (Roche), and data were collected using QuantStudio 12K Flex software. For RNA-Seq library preparation, sequencing, and analysis, see Supplemental Methods.

### Western blot analysis.

Cells were lysed with RIPA buffer. Protein (40 μg) was loaded and run on acrylamide gels. Proteins were transferred onto PVDF membranes incubated with the different primary antibodies (Supplemental Table 1). HRP-conjugated secondary antibodies against rabbit/mouse IgG were used. Signal was collected using Nine Alliance Q9 software.

### FISH.

Paraffin-embedded tissue samples were cut into 3 μm slices. After tissue was dried and deparaffinized, samples were treated for 10 minutes with hydrogen peroxide. Antigens were retrieved in a 0.5 M citrate pH 6 pressure bath for 20 minutes. Tissue slices were treated for 30 minutes with protease solution at 40ºC. Then, the RNAscope 2.5 HD detection kit protocol was followed according to the manufacturer’s instructions (322360, ACD). *LCOR* mRNA was detected using human *LCOR* RNAscope (1296771-C1, BioTechne). After signal detection, we continued with the IHF protocol.

### PLA.

Cells were seeded in coverslips and fixed with 4% PFA for 20 minutes. PLAs were performed according to the manufacturer’s instructions (NC.MR.100Red, Navinci). In brief, cells were blocked and incubated with rabbit (anti-HA, Abcam ab9110) and mouse (anti-ERα, Santa Cruz Biotechnology, SC-8002) primary antibodies at 4ºC overnight. Samples were then incubated with rabbit and mouse probes followed by enzymatic reactions. Cells were washed and mounted with DAPI mounting solution. Images were acquired using an upright Nikon Eclipse Ni-E fluorescence microscope. Data were processed and quantified using Fiji software.

### Statistics.

Sample sizes for animal studies were determined on the basis of pilot experiments or previous studies. Mice were randomized before cell injections and treatment allocation. Researchers were not blinded to the treatment groups during experiments or outcome analysis, since it was necessary to monitor each group. For all in vivo and in vitro experiments, independent biological replicates are indicated in the figure legends. Results are presented as the mean ± SEM. Normality was assessed using the Shapiro-Wilk test and homoscedasticity with Bartlett’s test. For comparisons between 2 groups, statistical significance was evaluated using an unpaired, 2-tailed *t* test (parametric data) or the Wilcoxon signed-rank test for paired data. For multiple-group comparisons, 1- or 2-way ANOVA was used. Pairwise group differences were assessed with Tukey’s honestly significant difference (HSD) test. For experiments with repeated measures over time, a mixed-design ANOVA was used. When ANOVAs were significant, post hoc pairwise comparisons of group means were conducted using estimated marginal means with Tukey’s adjustment. For survival analyses, differences between groups were assessed using the log-rank (Mantel-Cox) test. Correlation significance was assessed using the correlation coefficient (*R*) and the associated *P* value. For statistical significance, a *P* value of less than 0.05 was considered significant. All experiments were reproduced with at least 3 independent biological replicates unless otherwise specified in the figure legends.

### Study approval.

This study complied with all ethics regulations. PDOs and PDXs were established from tumor samples extracted by surgery from patients with primary BC at the Hospital del Mar and Vall d’Hebron University Hospital (Barcelona, Spain) following approval of the Clinical Research Ethics Committee of both institutions. PBMCs were obtained from consenting healthy bank donors of blood and tissue with prior approval BST ethics committee and the Hospital del Mar ethical committee. TMA samples were approved according to the ethics regulation of the Gipuzkoa clinical investigation committee (Gipuzkoa, Spain). All animal procedures in this study were approved by the Ethics Committee for Animal Research of Barcelona Biomedical Research Park (PRBB), regulated by the Department of Medi Ambient i Habitatge of Catalonia Government (Barcelona).

### Data availability.

Sequencing data are deposited in the NCBI’s Gene Expression Omnibus (GEO) database: AT3 and AT3-ER RNA-Seq (GSE292849); MCF-7 and MDA-MB-231 RNA-Seq with control and LCOR (GSE292767); and MCF-7 LCOR ChIP-Seq: LCOR-OE+vehicle, LCOR-OE+fulvestrant and LSKAA-OE (GSE292768). Previously published datasets that were analyzed are available under the original accession codes: GSE173839 (12) ISPY-2 RNA-Seq; EGAS00001004809 (42) BioKey scRNA-Seq; GSE55374 (43) Arthur et al.; GSE59515 (44) Turnbull et al.; GSE111563 (45) Selli et al.; GSE60789 Scan-B (50); and Papachristou et al. (74), Supplemental dataset 7 (4-OHT–treated MCF-7 cells RNA-Seq). The gene sets used for GSEA, GO, weighted gene co-expression network analysis, and gene set variation analysis (78) can be found in the MSigDB database, version 5.1, under Kyoto Encyclopedia of Genes and Genomes (KEGG) code M16004; Hallmarks 2020: M5906 and M5913; Reactome analysis (https://reactome.org/) and Elsevier Pathway Collection (https://www.elsevier.com/). METABRIC (accession number EGAS00000000083) and TCGA (accession number phs000569) RNA-Seq data are available at the cBioportal website (http://www.cbioportal.org/index.do). Transcriptomics data for BC cell lines are available in the CCLE (https://sites.broadinstitute.org/ccle/). All other data that support the findings of this study are present in the main article and the supplemental materials. Values for all data points in graphs are reported in the Supporting Data Values file. Raw immunoblot data are reported in the full unedited blot and gel images. The gating strategy for the different markers analyzed by FACS are provided in Supplemental Figure 8. For additional information of reactives and reagents used in this study refer to Supplemental Table 4. Data supporting the findings of this study are available from the corresponding author upon reasonable request. Additional methodological descriptions are in Supplemental Methods. 

## Author contributions

TCT conceived the concept of the study and supervised the project. JAP and TCT designed the study. JAP performed all in vitro, in vivo, and in silico experiments with the help of SBB, GSM, and IPN. HB and PTD performed the computational analysis. CC helped with the microscopy imaging and tissue microarray analysis. S Menendez performed IHC staining of tissue microarrays, and LC analyzed IHC and CD8 staining. J Albanell, LC, S Menendez, SS, TM, and MC provided tumor and blood human samples. JILV, S Manzano, MMC, and AU provided tissue microarray samples. RLB and LM analyzed ChIP-Seq and helped with scientific discussion. JAS performed the computational regression model. SDS and J Arribas provided the PDX. TCT and JAP wrote the manuscript. All authors discussed the results of the manuscript.

## Funding support

This work is the result of NIH funding, in whole or in part, and is subject to the NIH Public Access Policy. Through acceptance of this federal funding, the NIH has been given a right to make the work publicly available in PubMed Central.

- Asociación Española contra el cáncer (AECC) Proyectos Generales grant PRYGN234881CELI (to TCT).
- Generalitat de Catalunya grant SGR-22 00037 (to TCT).
- Ministry of Science, Innovation and Universities (MCIN) grant PID2023-147310OB-I00 (to TCT).
- Instituto de Salud Carlos III-FSE grant PI21/00020 (to TCT).
- Generalitat de Catalunya Agència de Gestió d’Ajuts Universitaris grant 2021 SGR 00776 and FEDER (to JAP).
- Roche, Synthon/Biondys, and Molecular Partners funding (to J Arribas).
- Postdoctoral AECC 2023 grant POSTD234709BLAS (to SB).
- AECC PhD Fellowship (PRDGI19007LOPE, to JILV).
- Sara Borrell Fellowship Instituto Carlos III (ISCIII) grant CD23/00059, co-funded by the European Union (EU) (to S Manzano).
- ISCIII grant PI21/01208, co-funded by the EU (to MMC).
- MCIN grants CNS2023-145020 and CPP2022-009535 (to MMC).
- Banco Bilbao Vizcaya Argentaria (BBVA) grant LEO23-2-10814-BBM-TRA-229 (to MMC).
- Ikerbasque Basque Research Foundation (to MMC).
- TMA material was funded by ISCIII grant PI20/01253, co-funded by the EU, and by a Basque Department of Health grant (2020111040) and a Sociedad Española de Oncología Médica (SEOM) Avon Fellowship 2020.
- Breast Cancer Research Foundation grant BCRF-25-008 (to JA).
- La Caixa Foundation grant HR22-00776 (to JA).
- World Cancer Research grant 22-0286 (to JA).
- MCIN grant FPU20/05388 (to SDS).
- BCRF grant R01GM141349 by the NIGMS (to LM).
- National Cancer Institute (NCI), NIH grant 1R01CA288742-01 (to LM).
- CIBERONC grants CB16/12/00241 and PI24/00192 ISCIII, co-funded by the EU (to J Albanell).
- Generalitat de Catalunya grant 2021 SGR 00776) (to J Albanell).

## Supplementary Material

Supplemental data

Unedited blot and gel images

Supporting data values

## Version 1 11/25/2025

In-Press Preview

## Version 2 01/16/2026

Electronic publication
