# Supplementary material for: Estrogen receptor signaling drives immune evasion and immunotherapy resistance in HR+ breast cancer
Source: J Clin Invest. 2025 Nov 25;136(2):e193153. doi: 10.1172/JCI193153 (PMC12807476; doi:10.1172/JCI193153)
Supplement: Supplemental data [file jci-136-193153-s077.pdf]

## Supplemental material

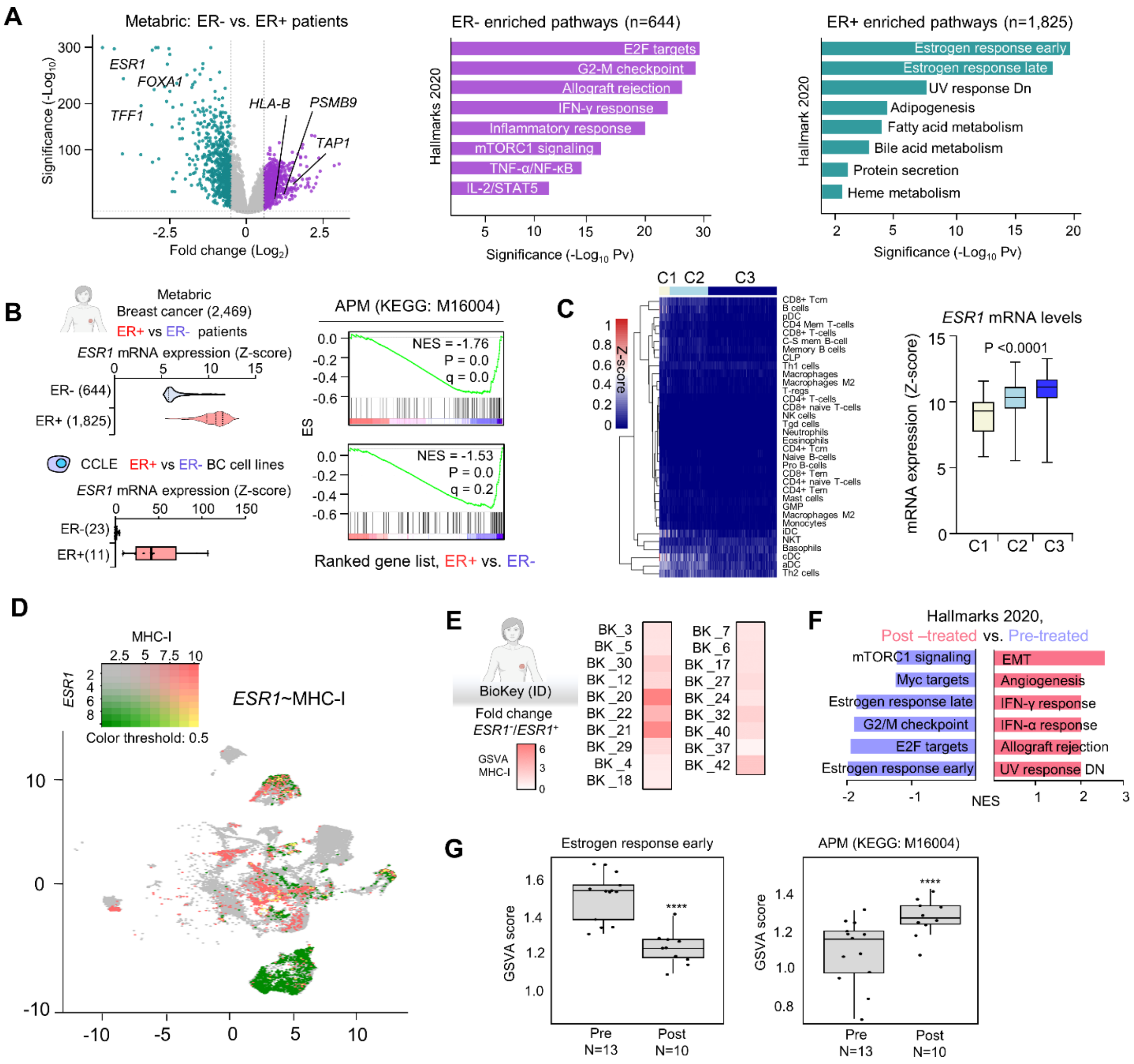


**Supplemental Figure 1. (A)** Volcano plot of the differential expressed genes between ER+/HER2- and ER-/HER2- patients from Metabric dataset. Top differentially enriched pathways (Hallmarks, 2020) in ER- (N=644) and ER+ (N=1,825) patients. Data is ranked by $Log_{10}$ P value. **(B)** GSEA of APM (KEGG: M16004) in ERα positive versus negative cell lines from CCLE and patients from Metabric dataset. Distribution of *ESR1* mRNA levels across patients and cell lines is shown as Z-score. **(C)** Computational immunophenotype clustering by deconvolution of HR+/HER2- Metabric dataset with xCell. Heat map represents clusters of patients based on the level of immune infiltration. *ESR1* mRNA levels across clusters of deconvolution immune phenotype. **(D)** UMAP representation of *ESR1* mRNA and GSVA of MHC-I signature co-expression in HR+ patients from BioKey HR+ cohort. **(E)** Fold change of GSVA MHC-I signature comparing ER- cells to ER+ within the same patient. **(F)** GSEA analysis of top enriched pathways (Hallmarks 2020) in Pre-treated patients and 2-weeks post letrozole treatment. Data is ranked based on NES. **(G)** Box plots of GSVA of APM (KEGG:16004) and estrogen response early (Hallmarks, M5906) including, pre and 2-weeks post treatment. Dots represent individual values. **** p<0.01 by Wilcoxon's paired test.

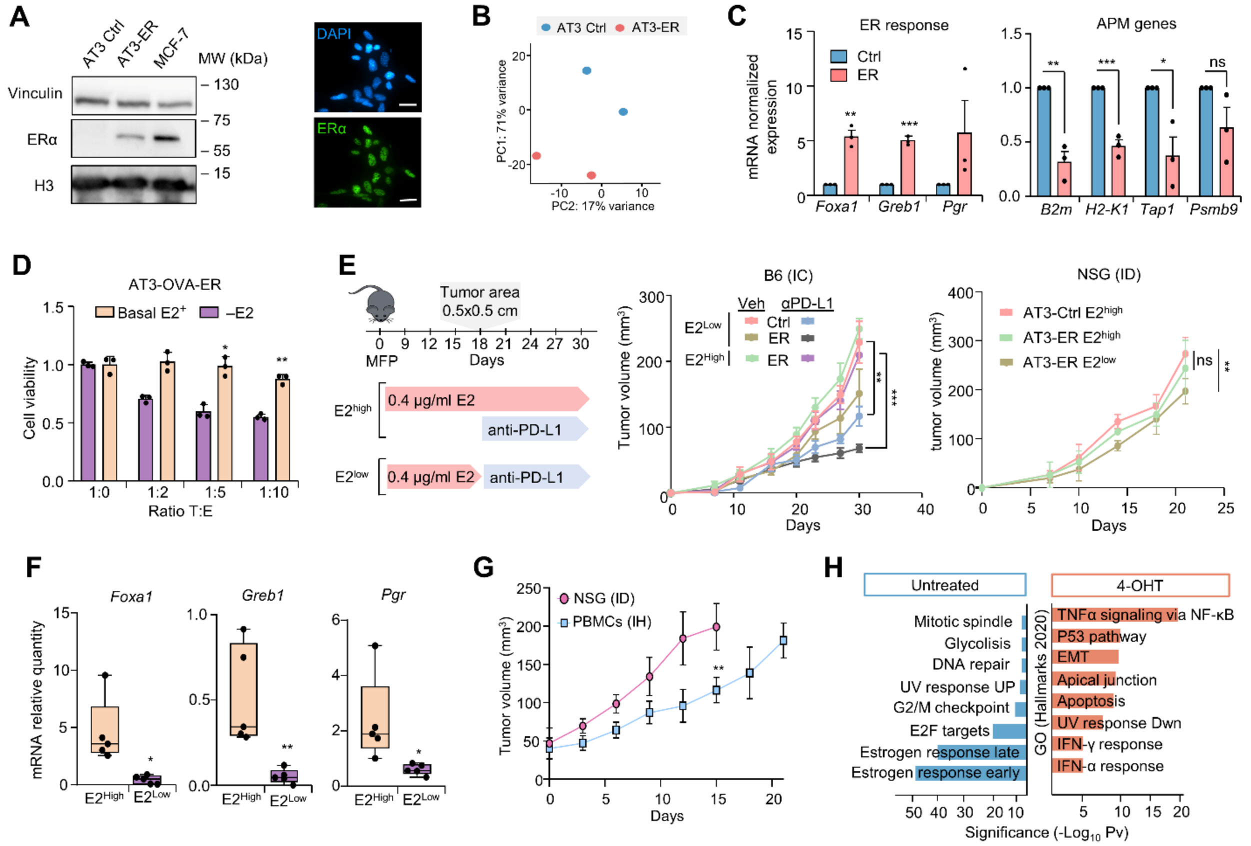


**Supplemental Figure 2. (A)** Western-blot of ERα over-expression in AT3 cells with MCF-7 protein extract as positive control. Representative IF image of ER nuclear staining together with DAPI from AT3 ER-OE. Scale bar, 20 μm. **(B)** Principal Component Analysis (PCA) of AT3 control and AT3-ER OE RNA-seq samples. N=2 independent biological replicates. **(C)** RT-qPCR analysis of ERα related transcripts (*Foxa1*, *Greb1* and *Pgr*) and APM genes (*B2m*, *H2-K1*, *Tap1* and *Psmb9*) in control and ER-OE AT3 cells. N=3 independent biological replicates. **(D)** CTL assay of AT3-OVA-ER cells cultured in basal media or charcoal stripped FBS media (-E2) at different tumor (T) and effector (E) ratio. **(E)** Growth curves of orthotopically implanted AT3 control or ER-OE tumors in B6 (IC) or NSG (ID) mice with vehicle ($E2^{Low}$) or E2 supplementation ($E2^{High}$, 0.4μg/ml) treated with anti-PD-L1 (7.5 μg/ml). N=5 tumor per condition. **(F)** RT-qPCR analysis of ERα related transcripts (*Foxa1*, *Greb1* and *Pgr*) in E2 high and low grown tumors. Box plot represents interquartile range with individual values. **(G)** Growth curves of orthotopically implanted MCF-7 tumors in immunodeficient NSG mice (ID) and immune humanized mice (IH). N=4 mice. **(H)** Gene enrichment analysis of upregulated (red) and downregulated (blue) pathways (MSigDB Hallmarks 2020) comparing MCF-7 control versus 4-OH-Tamoxifen treated transcriptome from public RNA-seq dataset (75). Pathways are ranked by $-Log_{10}$ P value. Data represent mean ± S.E.M. in **C**, **D**, **E** and **G**. Statistical significance; *p<0.05, **p<0.01, ***p<0.001, by two-way ANOVA in **C**, **D** and **F**. Mixed design ANOVA in **E** and **G**.

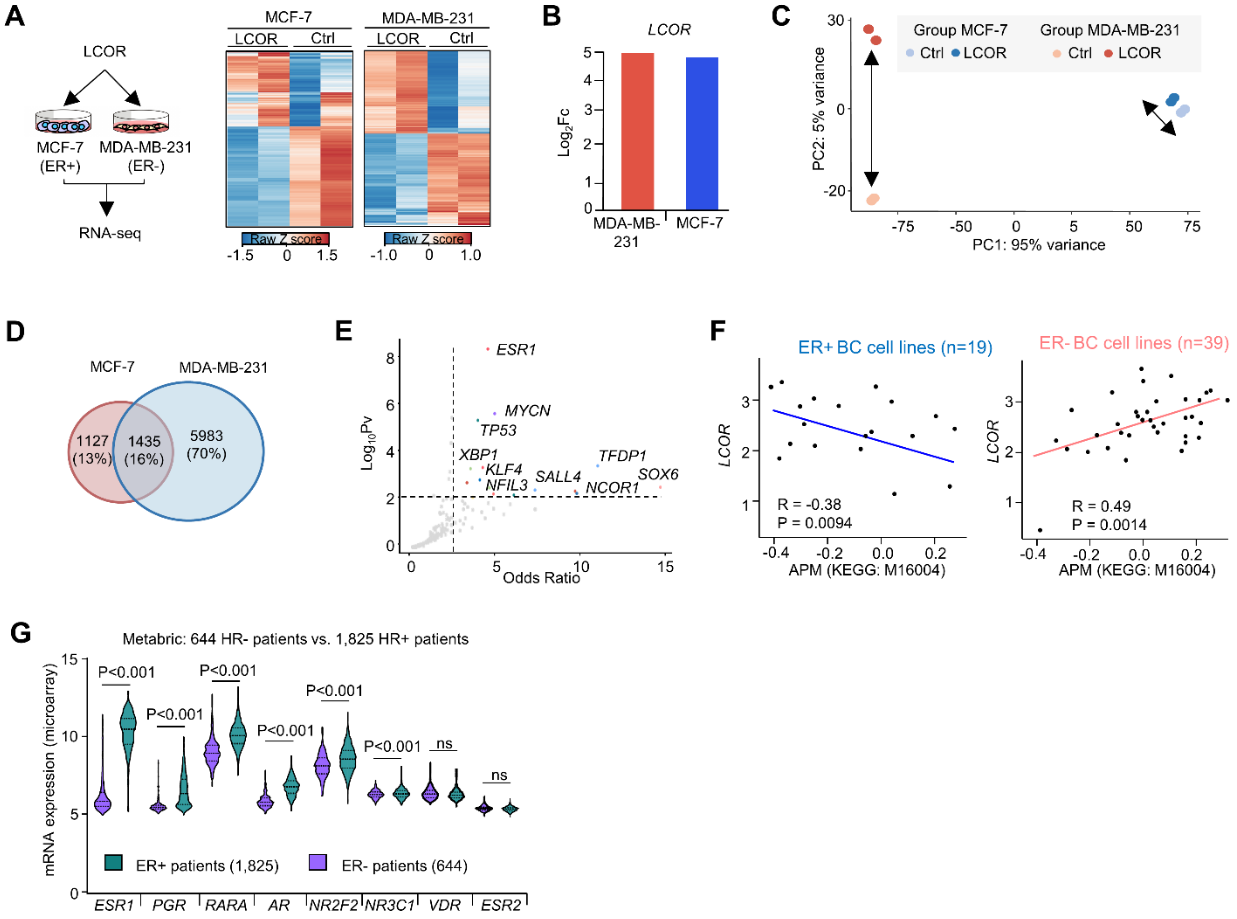


**Supplemental Figure 3. (A)** RNA-seq analysis of human HR+BC (MCF-7) and HR-BC cells (MDA-MB-231) with LCOR ectopic overexpression compared to control conditions. N=2 independent biological replicates. **(B)** $Log_2Fc$ of LCOR mRNA expression of MCF-7 and MDA-MB-231 cells from RNA-seq. **(C)** PCA of RNA-seq samples. Arrows indicate distance from Ctrl and LCOR-OE cells in PC1 (95% of variance) and PC2 (5% variance). **(D)** Venn diagram of overlapped significantly altered transcripts between MCF-7 LCOR-OE and MDA-MB-231 LCOR-OE cells to their respective control conditions. **(E)** ChIP-enrichment analysis from downregulated transcripts in LCOR-OE MCF-7 cells versus Control. **(F)** Pearson correlation of *LCOR* mRNA levels and APM signature (KEGG: M16004) of 19 HR+ and 39 HR- cell lines from CCLE. P value is calculated from R correlation. **(G)** mRNA expression of nuclear receptors between HR- and HR+ patients from Metabric dataset. P value of statistic test is shown.

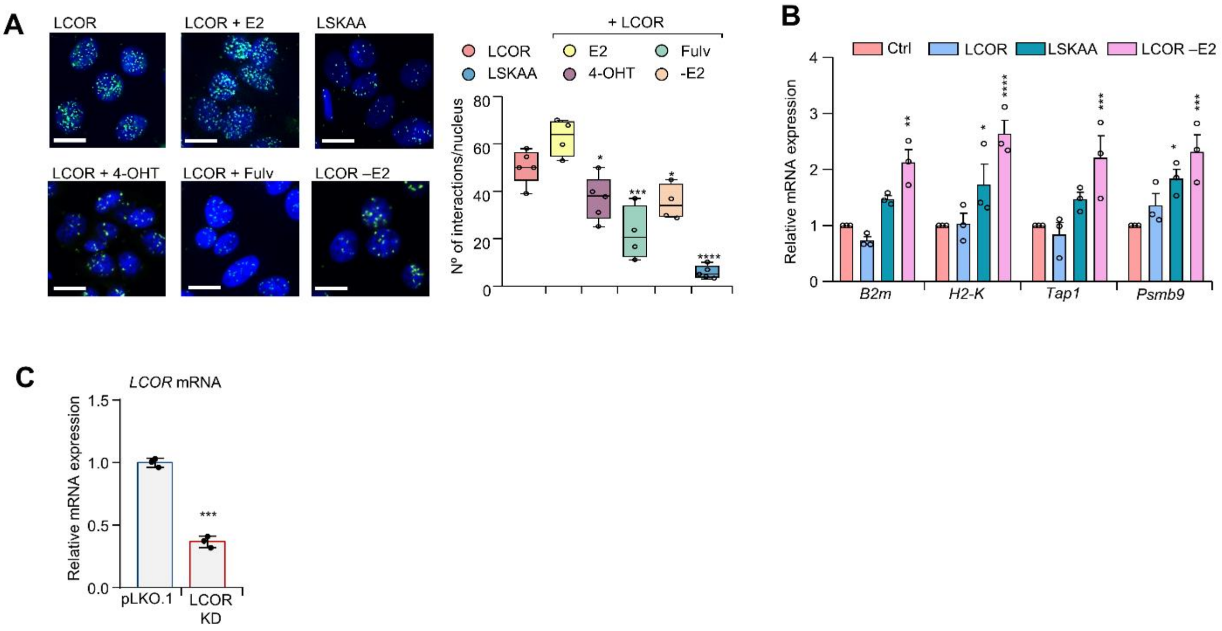


**Supplemental Figure 4. (A)** PLA for ERα and LCOR/LSKAA with HA tagged (anti-HA antibody) in MCF-7 cells treated with vehicle, E2, 4-OHT, Fulvestrant or charcoal FBS (-E2) during 48 hours. Representative microscopy images for the PLA green signal and DAPI nuclear staining. Quantification of PLA signal across conditions. Data represents the average PLA signal from 10 nuclei per condition. N=5 biological replicates. Boxplot represents interquartile range. **(B)** RT-qPCR analysis of Mhc-I/APM representative genes: *B2m*, *H2-K*, *Tap1* & *Psmb9*; in AT3-ER cells with control vector, LCOR-OE and LSKAA-OE treated with regular or -E2 media (charcoal FBS). N=3 independent biological replicates, data is represented as mean ± S.E.M. **(C)** RT-qPCR quantification of LCOR KD in MCF-7 cells using shRNA TRCN0000016306. N=3 independent biological replicates, data is represented as mean ± S.E.M. *$p<0.05$, **$p<0.01$, ***$p<0.001$, by one-way ANOVA test in **A** and **C**, two-way ANOVA in **B**.

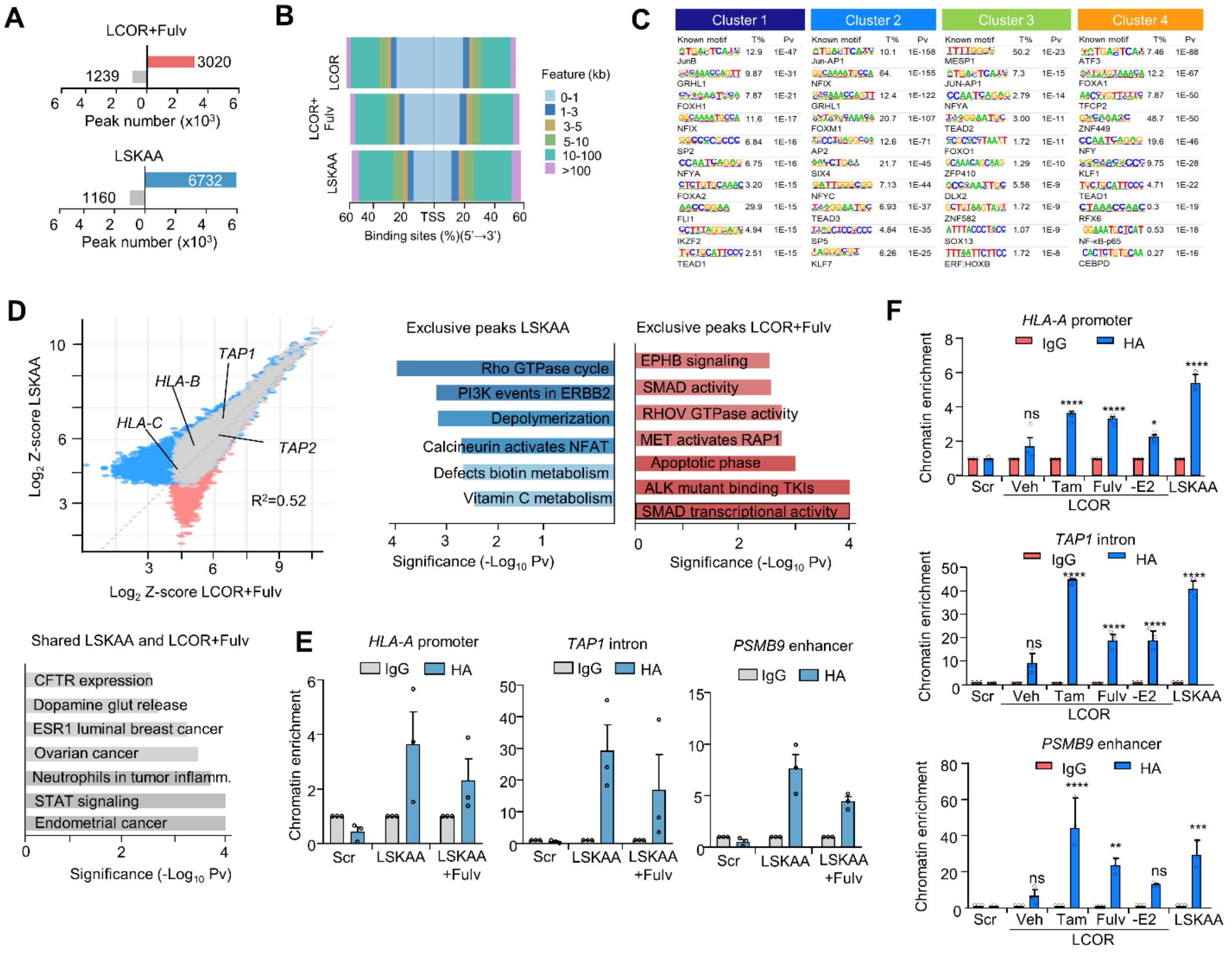


**Supplemental Figure 5. (A)** Bar plots representing total of gained and lost peaks in LCOR+Fulvestrant and LSKAA conditions compared to LCOR. **(B)** Genomic distance and percentage of LCOR and LSKAA binding sites relative to the transcription start site (TSS). **(C)** Top 10 DNA motifs from Homer analysis for the 4 different clusters defined by k-means clustering. Tables show motifs name, percentage of target (T%) and P value (Pv). Motifs are ranked according to P value. **(D)** Comparative analysis and scatter plot showing genome-wide changes of LCOR binding in LCOR+Fulvestrant and LSKAA MCF-7 cells. Red points denote sites induced by Fulvestrant, blue points denote sites induced by LSKAA; and grey points denote common peaks. $R^2$ is shown. Pathway analysis of the gained and lost and shared peaks ranked by $-Log_{10}$ P value. **(E)** ChIP-qPCR analysis of APM GRE: *HLA-A*, *TAP1* and *PSMB9* in MCF-7 scramble vector (Scr) or LSKAA-OE treated with vehicle or fulvestrant (1 µM). **(F)** ChIP-qPCR analysis of APM GRE*:* *HLA-A*, *TAP1* and *PSMB9* in MCF-7 cells overexpressed with scramble vector (Scr), LKSAA or LCOR construct treated with vehicle, 4-OH Tamoxifen (1 µM), Fulvestrant (1 µM) or E2 depletion (Charcoal medium). Data represents mean ± S.E.M. of three independent biological replicates; *p<0.05, **p<0.01, ***p<0.001, by two-way ANOVA test in **E** and **F**.

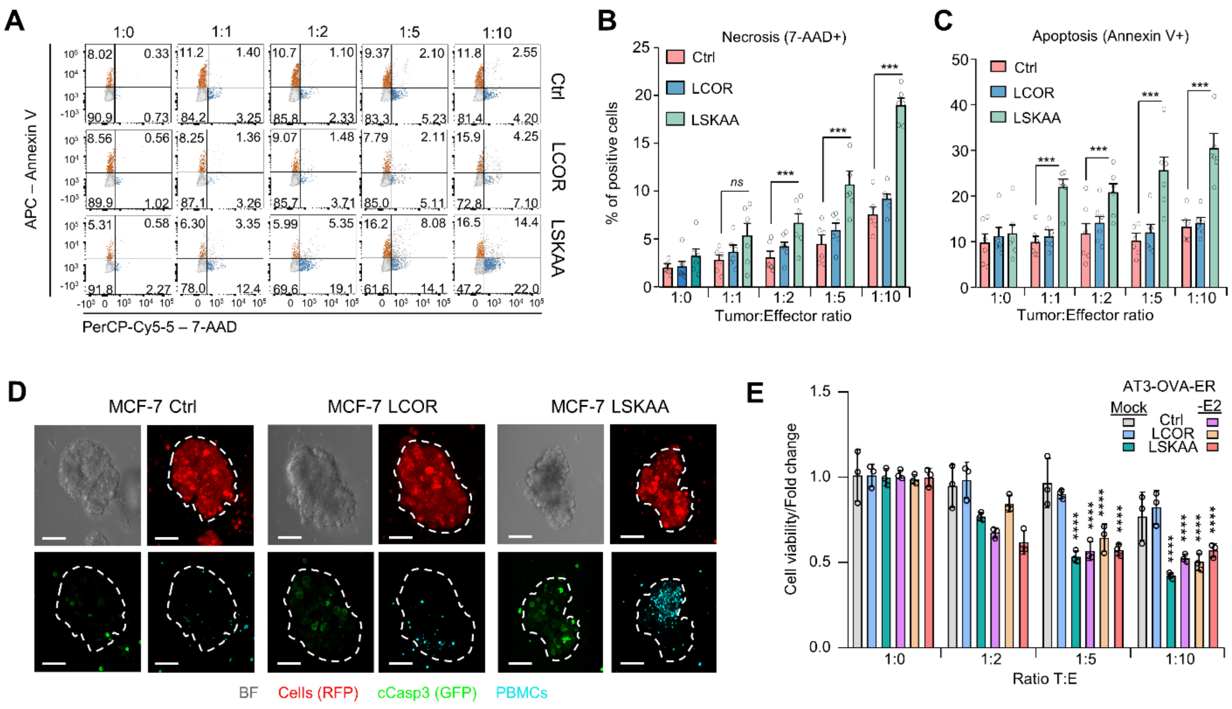


**Supplementary Figure 6. (A)** Flow cytometry plots showing Annexin V and 7-AAD staining of MCF-7 cells cocultured with PBMCs at different ratio for control, LCOR-OE and LSKAA-OE. The percentage of population within gate are shown. **(B)(C)** Percentage of necrotic cells (7-AAD+) **(B)** and apoptotic cells (Annexin V+) **(C)** from CTL of MCF-7 cells after 48h of coculture with PBMCs at the indicated conditions. Effector (E) and tumor (T) ratios are indicated. N=6 independent biological replicates. **(D)** Split channels of 3D co-culture of MCF-7 tumoroids and immune cells from **Figure 6B**. Tumoroid structures are visible through bright field (BF) and marked with dashed line. Scale bar, 10 µm. **(E)** Cell viability of AT3-OVA-ER cells after coculture with OT-I T cells. Effector (E):Tumor (T) ratios and conditions are depicted. N=3 independent biological replicates. Data represents mean ± S.E.M.; Statistical significance: *p<0.05, **p<0.01,***p<0.001, by two-way ANOVA test in **B**, **C** and **E**.

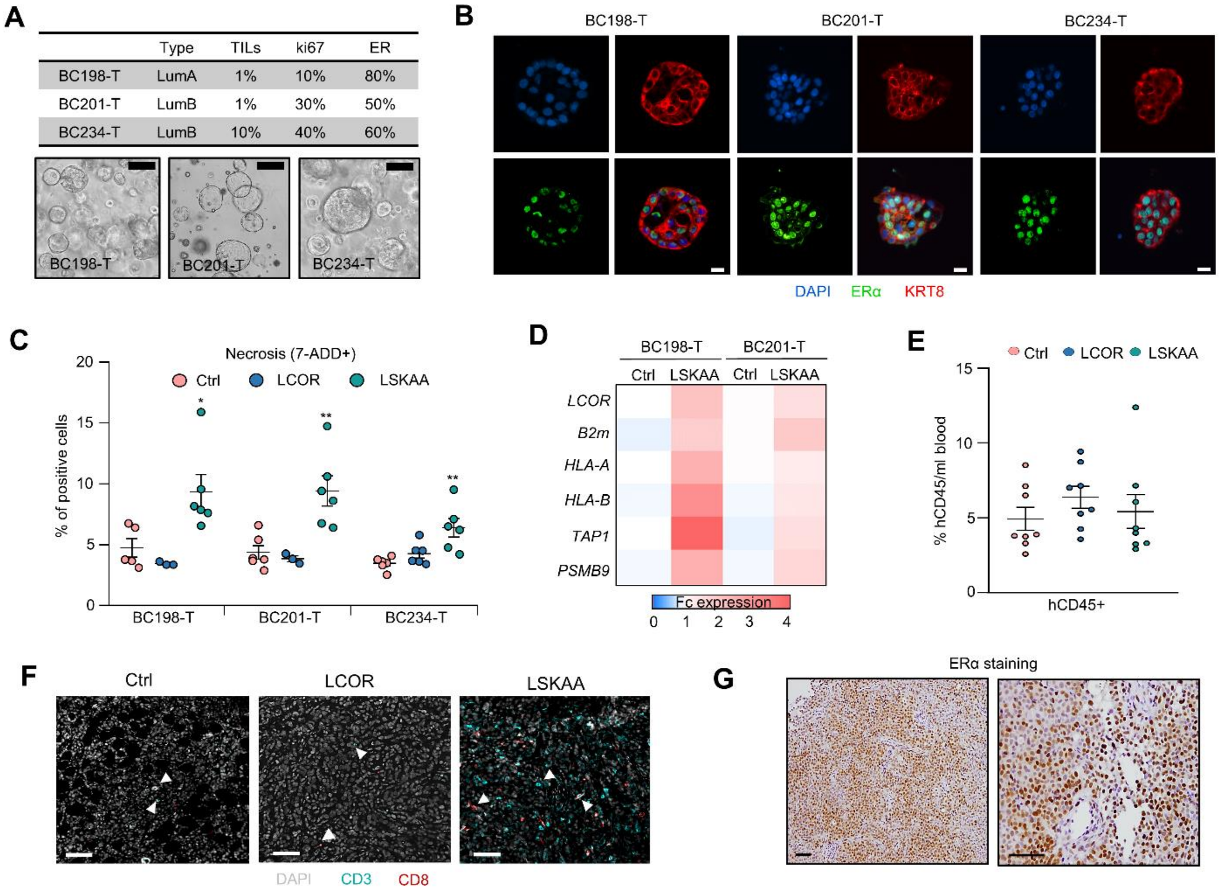


**Supplemental Figure 7. (A)** Representative images of HR+ BC PDOs from three patients with different clinic-pathological features listed in table. **(B)** Confocal microscopy images of HR+ BC PDOs stained with ERα (FITC), KRT8 (RFP) and DAPI. Scale bar 10 µm. **(C)** Flow cytometry analysis of cell death: necrosis (7-AAD); from control, LCOR-OE and LSKAA-OE PDOs cocultured with autologous PBMCs. N=5 independent biological replicates. Data represents mean ± S.E.M. Statistical significance; *p<0.05, **p<0.01, ***p<0.001, by two-way ANOVA test. **(D)** RT-qPCR analysis of MHC-I/APM gene expression levels from HR+ BC PDO's transduced with control plasmid or LSKAA-OE. Color scale represent $Log_2Fc$ of expression. **(E)** Flow cytometry analysis of mice blood after 3 weeks of human PBMCs inoculation measured as percentage of human CD45+ cells from mouse CD45+ cells. Data represents mean ± S.E.M. **(F)** Tumor tissue immunofluorescence of CD3+ and CD8+ immune cells from control, LCOR-OE and LSKAA-OE MCF-7 tumors in mice inoculated with PBMCs. Arrows show immune cell populations. Scale bar, 100 µm. **(G)** IHC of ERα expression from PDX173. Scale bar, 100 µm. Cells were gated as shown in supplemental figure **8A** for **C** and supplementary **8B** in **E**.

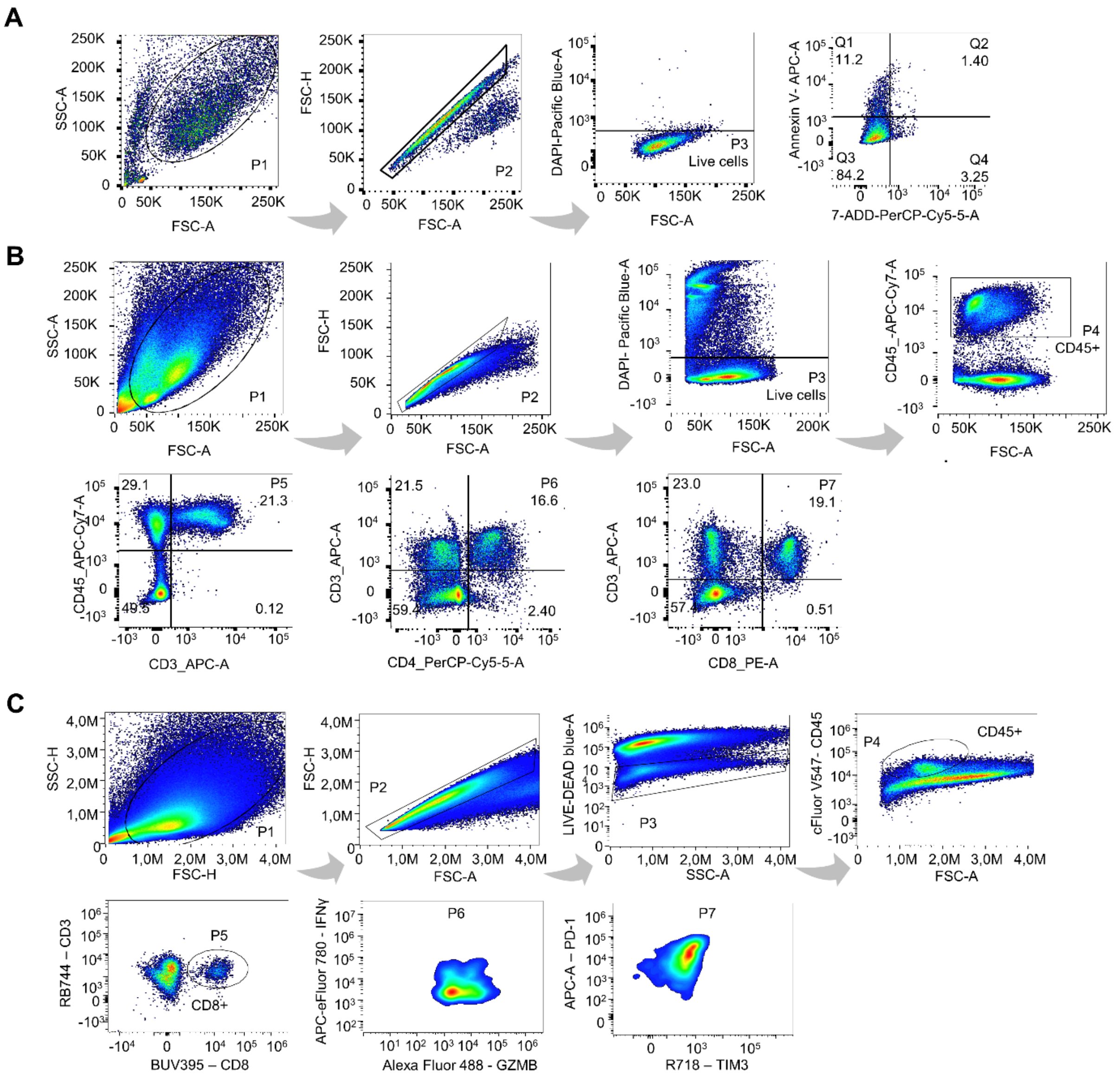


**Supplemental Figure 8. (A)** Gating strategy used to define live single cells population (P3) for all in vitro models used in this study; and to distinguish cell death through apoptosis (Annexin V+, Q1) and necrosis (7-ADD+, Q4). **(B)** Gating strategy to define specific immune cell subsets in in vivo studies: immune infiltration (P4, CD45+), lymphoid compartment (P5, CD3+/CD45+), CD4+ T cells (P6, CD3+/CD4+), CD8+ T cells (P7, CD8+/CD3+). **(C)** Gating strategy to define specific immune cell subsets in in vivo studies: T cell phenotype. Immune infiltration (P4, CD45+), CD8+ T cells (P5, CD3+/CD8+), activation (P6, GZMB+/IFN-γ+); and exhaustion (P7, PD1+/TIM3+).

**Supplemental material and methods**

**ChIP-seq analysis**

Fasq files quality were checked with FastQC software. Reads were aligned with the bowtie2 mapper to release 27 of Homo sapiens Gencode version of the genome (GRCh38/hg38 assembly). Quality of the mapped files (BAM format) was checked with QualiMap. Peaks on individual samples were identified using the MACS2 software (narrow peaks with q<0.1 were initially selected). Overlap of peaks across biological replicates were retrieved using the Bedtools. To visualize the distribution of chromatin accessibility and transcription factor binding across genomic regions, we generated a heatmap using deepTools v3.5.5 (PMID: 27079975). The computeMatrix scale-regions function was employed to process BigWig files corresponding to different experimental conditions relative to a set of predefined genomic regions. The generated matrix was then visualized using plotHeatmap, specifying a Blues colormap, hierarchical clustering of regions, and labeled experimental conditions. For comparative peak intensity analysis, scatter plots were generated in R using the GenomicRanges, rtracklayer, and ggplot2 packages. BED files containing peaks from two conditions were imported and merged, filtering for standard chromosomes and removing non-canonical contigs. Shared and condition-specific peaks were identified by finding overlaps within a 100 bp tolerance. Signal intensities were extracted from BigWig files using overlaps with peak regions, and the log2-transformed values were plotted. Regression lines were added to assess correlation. Gene annotation was performed using ChIPseeker and TxDb.Hsapiens.UCSC.hg38.knownGene, identifying transcription start site (TSS)-associated peaks. For motif enrichment analysis, we utilized HOMER (Hypergeometric Optimization of Motif EnRichment). BED files corresponding to different peak clusters were processed using findMotifsGenome.pl, specifying the hg38 genome as the reference.

**RNA-seq analysis**

Library was prepared with poly-A library sequencing and samples were sequenced using the Illumina Hi-Seq 2500 platform with 1x50 bp setting at Centre of Genomic Regulation (CRG). Fastq files quality was check with FastQC software. Estimation of ribosomal RNA in the raw data was obtained with riboPicker. Quality reads were aligned with the STAR mapper to the Homo sapiens genome

GRCh38/hg38. The raw amount of reads per gene and sample was obtained with STAR (quantMode TranscriptomeSAM GeneCounts). The R/Bioconductor package DESeq2 v.1.30.1 was used to assess differential expression between experimental groups (Wald statistical test plus false discovery rate, FDR, correction). Genes whose sum of raw counts across all samples was <1 were discarded.

**Clinical dataset analysis**

ISPY-2 clinical trial (no. GSE173839) (12) patients (n=50) were classified into Responder or non-Responder group depending on pCR state. Differential gene expression analysis was performed for both groups from available transcriptomic processed data. BioKey clinical trial (no. EGAD00001006608) included scRNA-seq transcriptomic data of Pre and on-treatment to ICB in HR+ BC (n=15) (42). Metabric (n=1,825) and TCGA (n=776) public datasets were obtained from cBioportal website. Scan-B transcriptomic data was obtained from GSE60789 (50). HR+ patients (n=2,423) were stratified according to *ESR1/PGR/NR2F2* tercile expression prior to *LCOR* median normalized expression. Anti-ER cohorts were extracted from public available datasets: GSE55374 (43), GSE59515 (44) and GSE111563 (45). Data was analyzed comparing post-treated patients after 2 weeks of letrozole versus pre-treated.

**Gense set enrichment analysis (GSEA), Gene ontology (GO) and Chromatin enrichment analysis (ChEA)**

GSEA correlated signatures between two conditions using 1,000 permutations of the labelled phenotype. Data was analyzed using P value, q value and normalized enrichment score (NES). Genes were ranked according to $r(_g)$ = sign $(Fc_g)(1\text{-}P_g)$, where r(g) is the enrichment score for each gene, $FC_g$ reflect the median fold change between two conditions and $P_g$ represent P value of Wilcoxon rank-sum test. Transcriptomic datasets were interrogated with different signatures: human and mouse hallmarks gene sets; the human Kyoto Encyclopedia of Genes and Genomes; human Reactome and Elsevier collection for ChIP-seq differential analysis. Downregulated genes in MCF-7 LCOR vs Ctrl were interrogated for enriched transcription factors using ChEA.

**Gense set variation analysis (GSVA)**

The GSVA package was used to establish an enrichment score representing the enrichment of signatures across patients (77). GSVA was applied using the following datasets: BioKey clinical trial, letrozole treatment cohorts (43–45) and transcriptomic data from 39 ER− and 19 ER+ breast cancer cell lines from the CCLE. Datasets were interrogated with the following gene signatures: estrogen response early (Hallmarks, M5906), APM signature (KEGG: M16004) and MHC-I (*B2M, HLA-A, HLA-B, HLA-C, TAP1, TAP2, TAPBP, PSMB8* and *PSMB9*).

**Deconvolution and immunophenotype analysis**

For estimation of immune infiltrating cells, the xCell algorithm was used on Metabric HR+ BC data. A non-hierarchical k means cluster analysis of three clusters based on Euclidean distance was used. *ESR1* expression was measured in the obtained 3 clusters of immune phenotypes.

**Regression model for interaction**

GSVA was used to generate single sample signatures from the Log2 transformed (Log2(x+1)) FPKM counts from TGCA HR+ BC samples for APM_1 (KEGG legacy, M16004), APM_2 (*HLA-A, HLA-B, HLA-C, TAP1, TAP2, TAPBP, PSMB8, PSMB9, B2M*) and Cytolytic score (51) signatures. GSVA was run using gaussian kernel and default parameters. Interaction of LCOR with the different NR for the three signatures was calculated using a normal linear model such that Score~geneA*geneB where score represents the expression levels of a predefined gene signature (APM_1, APM_2 and cytolytic score) and gene A correspond to LCOR and gene B the different NR assessed. All models were fitted using the lm() function in R (version 4.3.3). Rms was used to generate the model (ols functions) and plot the data. The significance of the interaction terms was evaluated using P values derived from the model summary, which is depicted in the figure and log10.

**Weighted Gene Co-expression Network Analysis (WGCNA)**

To investigate the potential relationship between LCOR, APM pathway genes, and the response to hormonal therapy, we performed a Weighted Gene Co-expression Network Analysis (WGCNA) to letrozole treatment cohort (43). WGCNA allow to identify gene modules based on co-expression

patterns to discover indirect regulatory relationships by clustering genes with similar expression profiles into modules. The analysis was conducted independently for each treatment timepoint to capture temporal dynamics in gene co-expression networks. Soft-thresholding was applied to construct scale-free networks, and modules were identified using hierarchical clustering based on topological overlap. From these modules, we specifically extracted the one containing LCOR and performed gene ontology pathway analysis, assessing their potential co-regulation.

**Single-cell RNA-seq analysis**

Available count data from single-cell RNA-seq (42) were downloaded in the form of an R-readable RDS file, along with corresponding metadata. Both cohorts 1 (treatment-naïve patients) and 2 (on-treatment patients receiving anti-PD1) were considered (total n = 29). Count data were pre-processed and analyzed with the Seurat R package. Low-quality cells were first filtered in order to have a number of features <6,000 and >200, with <15% mitochondrially derived genes. The remaining analysis was performed on paired samples from ER+ patients (n = 19) cancer cells alone. Counts were then normalized with the modelling framework for normalization method ‘sctransform v2’. The ‘addModuleScore’ Seurat function was used to calculate a score for each cell for estrogen response early (Hallmarks, M5906). The uniform manifold approximation and projection dimensional reduction technique was run, and cells were colour coded according to that score. Percentage of expressing cells for the given signature in each group was calculated and Wilcoxon’s paired test was applied to check significant changes.

**Supplemental table 1 - Antibodies**

| Antibodies | Source | ID |
|---|---|---|
| IF/IHC: Estrongen Receptor alpha (D-12) | Santa cruz | sc-8005 |
| IF: Ms mAb CD8 alpha | Abcam | AB17147 |
| IF: Rb pAb CD3 | Abcam | AB5690 |
| IF: mAb mouse CD4 | Thermo Fisher | 4SM95 |
| IF: Rb mAb mouse CD8a | Abcam | AB217344 |
| IF: Rb pAB to HA tag | Abcam | AB9110 |
| IF: Rb mAb to cytokeratine 8 | Abcam | AB53280 |
| IF: Goat anti-Rabbit IgG (H+L) cross-Adsorbed secondary antibody AF 555 | Invitrogen | A21428 |
| IF: Goat anti-Rabbit IgG (H+L) cross-Adsorbed secondary antibody AF 647 | Invitrogen | A21245 |
| IF: Goat anti-Mouse IgG (H+L) Cross-Adsorbed secondary antibody AF 555 | Invitrogen | A28180 |
| IHC: ImmPRESS HRP Goat Anti-Mouse IgG Polymer Kit Peroxidase | Vector Labs | MP-7452 |
| FC: PE anti-human CD3 | Biolegend | 980008 |
| FC: PerCP anti-human CD4 | Biolegend | 300528 |
| FC: APC/Cyanine7 anti-human CD45 | Biolegend | 368516 |
| FC: APC anti-human CD8 | Biolegend | 344722 |
| FC: PE-cyanine5 anti-human HLA-ABC | Invitrogen | 15-9983-42 |
| FC: PE anti-human B2-microglobulin | Biolegend | 316305 |
| FC: APC anti-Mo OVA257-264 (SIINFEKL) peptide bound to H-2Kb | Invitrogen | 2384290 |
| FC: PercP anti-mouse CD4 | Biolegend | 100434 |
| FC: APC anti-mouse CD3 | Biolegend | 100236 |
| FC: PE anti-mouse CD8a | Biolegend | 100708 |
| FC: APC/Cyanine7 anti-mouse CD45 | Biolegend | 103116 |
| FC: cFluor v547 anti CD45 | Cytek | R7-20571 |
| FC: BUV395 CD8 | BD Bioscience | 565968 |
| FC: BV750 CD4 | Biolegend | 100467 |
| FC: AF488 GZMB | Biolegend | 396423 |
| FC: RB744 CD3 | BD Bioscience | 570649 |
| FC: PE-CF594 CD69 | BD Bioscience | 562455 |
| FC: APC PD1 | Biolegend | 135210 |
| FC: R718 TIM3 | BD Bioscience | 568832 |

| | | |
|---|---|---|
| FC: APC eFluor780 IFNγ | Thermo | 47-7311-80 |
| IP: Normal Rabbit IgG | Cell signaling | 2729 |
| IP: Rb pAB to HA tag | Abcam | AB9110 |
| WB: Rb Estrogen Receptor alpha | Cell signaling | 8644 |
| WB: Rb pAB to Ms IgG (HRP) | Abcam | ab6728 |
| WB: Goat pAB to Rb IgG (HRP) | Abcam | AB6721 |
| WB: mouse monoclonal anti-vinculin (Clone 7F9) | Santa cruz | sc-73614 |
| WB: Rb pAb to Histone H3 | Abcam | ab1791 |
| In vivo: *inVivo*MAB anti-mouse PD-L1 (B7-H1) | Bioxcell | BE0101 |
| in vivo: InVivoMAb rat IgG2b isotype control, anti-keyhole limpet hemocyanin | Bioxcell | BE0900 |

**Supplemental table 2 – Plasmid constructs**

| Plasmid | Source | ID |
|---|---|---|
| Lentiviral overexpressing construct pLEX-*LCOR-LSKAA*-HA | (Cellà-Terrassa et al., 2017) | N/A |
| Lentiviral overexpressing construct pLEX-*LCOR*-HA | (Cellà-Terrassa et al., 2017) | N/A |
| Lentiviral overexpressing construct pLEX-HA | (Cellà-Terrassa et al., 2017) | N/A |
| Lentiviral overexpressing construct pLEX-OVA-IRES-mCherry | Y. Kang lab | N/A |
| shRNA lentiviral plasmid pLKO.1 | Merck | SHC001 |
| shRNA lentiviral plasmid pLKO.1 TRCN0000016306 | Merck | TRCN0000016306 |
| VSV.G | Cellà-Terrassa et al 2017 | Addgene: 14888 |
| pCMV-R8.91 | Cellà-Terrassa et al 2017 | Addgene: 2221 |
| Lentiviral overexpressing construct pLEX-*Lcor*-HA | This study | N/A |
| Lentiviral overexpressing construct pLEX-*Lskaa*-HA | This study | N/A |
| Piggybac Transposase-TRE construct *LCOR*-HA | PB-TRE-dCAS9 VPR, addgene | 63800 |
| Piggybac Transposase-TRE construct *LSKAA*-HA | PB-TRE-dCAS9 VPR, addgene | 63800 |
| Piggybac Transposase-TRE construct HA | PB-TRE-dCAS9 VPR, addgene | 63800 |

| | | |
|---|---|---|
| Lentiviral overexpressing construct ZipGFP-Casp3 | ZipGFP-Casp3, addgene | 81241 |
| Lentiviral overexpressing pHAGE-*ESR1* | addgene | 116737 |

**Supplemental table 3 – ChIP qPCR primers**

| Oligo | Sequence (5' – 3') |
|---|---|
| PSMB9_Enh_fw | CTGGCCAGAGGAATGAAGAA |
| PSMB9_Enh_rev | GCCCTTAAGGCATGTCACAC |
| TAP1_intr_fw | ATAGTCTGGGCAGGCCACTT |
| TAP1_intr_rev | TCGGAAAGTCCCAGGAACAG |
| HLAA_pr_fw | CGTAGGTTGGGAGAGGGAGA |
| HLAA_pr_rev | AAGGCGGTGTATGGATTGGG |

**Supplemental table 4 - Reagents**

| Chemical, peptides and recombinant proteins | Source | ID |
|---|---|---|
| Cell tracker deep red | Thermo Fisher | C34565 |
| Cell tracker green CMFDA | Thermo Fisher | C7025 |
| Hyaluronidase from bovine testes | Merck | H3506-500mg |
| Collagenase A | Merck | 10103578001 |
| Dispase | Merck | D4693 |
| DnaseI | Merck | D5025 |
| Protein G Dynabeads | Thermo Fisher | 10003D |
| AgeI-HF | NEB | R3552S |
| SpeI-HF | NEB | R3133S |
| NheI-HF | NEB | R3131S |
| Matrigel non-growth factor reduced | Corning | 354234 |
| ACK lysing buffer | Gibco | A10492-01 |
| FcγIII/II receptor CD16/CD32 | Biolegend | 101302 |
| True-Stain Monocyte blocking antibodies | Biolegend | 426102 |
| fixable viability dye live/dead blue | Invitrogen | L23105 |
| Foxp3/Transcription Factor kit | Invitrogen | 005-52300 |
| CD8a$^{+}$ T cell isolation kit | Milteny | 130-104-075 |
| DAPI mounting solution | SouthernBiotech | 0100-20 |
| ImmPress IgG polymer peroxidase kit | VectorLabs | MP-7452 |
