# Supplementary figures and images for: Estrogen receptor signaling drives immune evasion and immunotherapy resistance in HR+ breast cancer
Source: J Clin Invest. 2025 Nov 25;136(2):e193153. doi: 10.1172/JCI193153 (PMC12807476; doi:10.1172/JCI193153)

Full unedited blot for Supplemental Figure 2A

ER $\alpha$

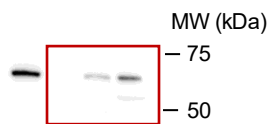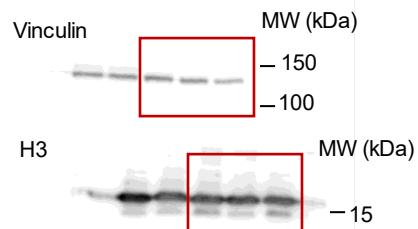

Supplement: Unedited blot and gel images [file jci-136-193153-s078.pdf]
